# Prokaryotic PfaB is a terminal acyltransferase that determines the final PUFA product

**DOI:** 10.1002/pro.70497

**Published:** 2026-02-12

**Authors:** Nahuel Lofeudo, Aurora Martín, Mateo Jácome, Xia Wan, María Lucas, Gabriel Moncalián

**Affiliations:** ^1^ Department of Molecular Biology Institute of Biomedicine and Biotechnology of Cantabria (IBBTEC), University of Cantabria‐CSIC Santander Spain; ^2^ Department of Functional Genomics Oil Crops Research Institute of Chinese Academy of Agricultural Sciences Wuhan China; ^3^ Key Laboratory of Biology and Genetic Improvement of Oil Crops Ministry of Agriculture Wuhan China

**Keywords:** acyltransferase, DHA, EPA, fatty acid synthase, polyketide synthase, polyunsaturated fatty acid

## Abstract

Omega‐3 polyunsaturated fatty acids (PUFAs) are essential for human health due to their numerous beneficial biological properties. These compounds are synthesized in marine bacteria and eukaryotic microalgae by PUFA megasynthases (Pfas), which are evolutionarily related to fatty acid synthases (FAS) and polyketide synthases (PKS). In FAS, PKS, and PUFA synthases, the acyltransferase (AT) domain plays a critical role in condensation reactions by loading starter or extender units into the acyl carrier protein (ACP) domain. PfaB, a component of PUFA megasynthases, harbors a pseudo‐ketosynthase (KS′) domain and an AT domain. In this study, we show that PfaB determines the final PUFA product, as demonstrated by in vivo assays in *Escherichia coli* using the DHA‐producing *Moritella marina* and the EPA‐producing *Shewanella baltica*. In vitro biochemical assays confirm that PfaB exhibits acyltransferase activity, with distinct substrate specificity from the AT domain of PfaA. Finally, we report the crystal structure of PfaB from *S. baltica*, representing the first structurally resolved AT domain within a PUFA megasynthase. Molecular docking analyses suggest that specific residues may contribute to differences in substrate recognition and specificity. Together, these findings show that PfaB acts as the terminal acyltransferase, providing new insights into its functional role in PUFA biosynthesis, and advancing our understanding of its mechanism and ligand interactions.

## 1 INTRODUCTION

**FIGURE 1:**
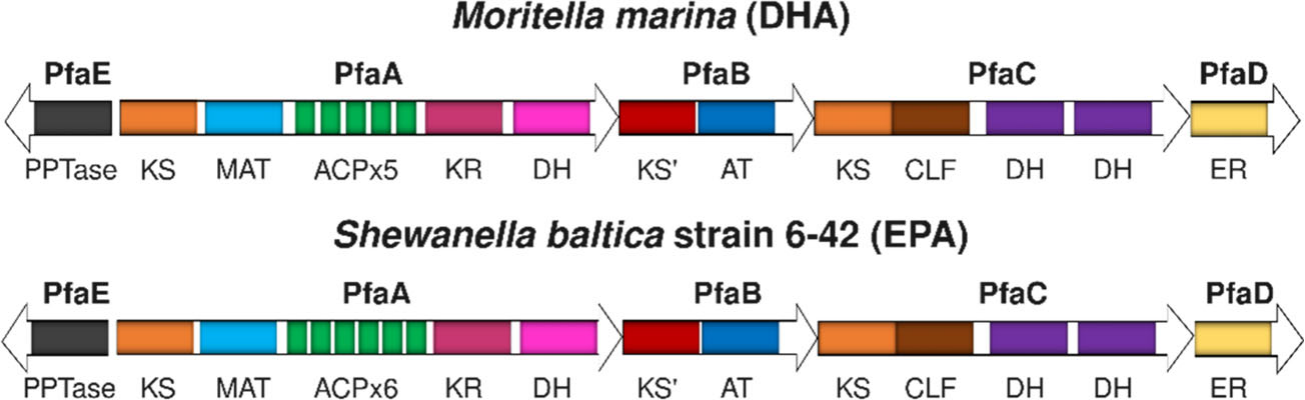
Domain organizations of *M. marina* and *S. baltica* PUFA synthases. KS, ketosynthase; MAT, malonyl‐CoA acyltransferase; ACP, acyl carrier protein; KR, ketoreductase; DH, dehydratase; AT, acyltransferase; CLF, chain length factor; and ER, enoyl reductase.

Omega‐3 polyunsaturated fatty acids (PUFAs) such as docosahexaenoic acid (DHA; C22:6) or eicosapentaenoic acid (EPA; C20:5) play key roles in human health (Swanson et al., 2012). Interestingly, PUFAs can also be synthesized directly from malonyl‐CoA in marine gammaproteobacteria and thraustochytrids by PUFA megasynthases (Pfas), a system with promising biotechnological applicability (Jovanovic et al., 2021). Pfas resemble mammalian fatty acid synthases (FAS) and microbial polyketide synthases (PKS). They are composed of three multidomain polypeptides in thraustochytrids and myxobacteria, or four in marine gammaproteobacteria (Shulse & Allen, 2011). In the latter, the gene cluster *pfaA‐D* encodes the core complex. *pfaE*, located elsewhere, encodes a phosphopantetheinyl‐transferase that activates the acyl carrier protein (ACP) loading 4‐phosphopantetheine (PPT). PUFA synthases typically contain 5–9 tandem ACP domains with 85%–96% sequence identity, with higher ACP copy number correlating with increased productivity (Hayashi et al., 2016; Jiang et al., 2008). Their enzymatic domains include condensing (acyltransferase [AT], ketosynthase [KS]) and modifying (ketoreductase [KR], dehydratase [DH], enoyl‐reductase [ER]) activities (Figure 1). The AT domain transfers the acyl extender units to ACP, while KS allows the chain elongation. Modifying domains reduce the intermediates to fully or partially saturated fatty acids.

In *Moritella marina*, PfaA‐ACP domains can either self‐malonylate or be modified by the PfaA malonyl acyltransferase (MAT) domain (Santín & Moncalián, 2018). Subsequently, PfaA‐KS decarboxylates malonyl‐ACP to produce acetyl‐ACP, which is then condensed with another malonyl‐ACP. Therefore, 3‐oxo‐butanoyl‐ACP is produced in the starting condensing step; this intermediate is fully reduced to butanoyl‐ACP by the modifying domains. The cycle is repeated seven times to produce C18:4. Final chain elongation is performed by the PfaC‐KS‐CLF didomain (Santín et al., 2020).

Contrary to FAS, gammaproteobacterial PUFA synthases contain repeated domains (KS, AT, ACP, and DH). Two AT domains are present: one in PfaA with MAT activity, and another in PfaB with unknown specificity. PfaB also includes a pseudo‐KS (KS′) domain, which is inactive due to the absence of the conserved Cys‐His‐His catalytic triad (Santín et al., 2020). In contrast, thraustochytrid (i.e., *Auriantiochytrium* sp., *Schizochytrium* sp., or *Thraustochytrium* sp.) PfaB proteins harbor an active KS‐CLF didomain, along with AT and ER domains. PfaB determines the final PUFA chain length (Orikasa et al., 2009) and exhibits hydrolase activity against long‐chain acyl substrates (Almendáriz‐Palacios et al., 2021; Hayashi et al., 2020), suggesting a role in off‐loading the final product. Furthermore, its low affinity for malonyl‐CoA (Santín & Moncalián, 2018) supports its involvement in the final steps of PUFA biosynthesis.

To elucidate the function of PfaB in proteobacterial PUFA synthesis, we have characterized its activity both in vivo and in vitro, confirming that PfaB retains acyltransferase function. We also report the first solved structure of a PfaB protein from the EPA‐producing *Shewanella baltica* strain 6–42. Structural analysis reveals a flexible KS′ domain with weak electron density. The AT domain adopts the characteristic α/β hydrolase and ferredoxin‐like subdomains, despite sharing only ⁓15% sequence identity with other AT solved structures. Molecular docking and structural comparisons with predicted models of PfaA‐MAT and PfaB from the DHA‐producing *M. marina* reveal variations in the substrate‐binding channel. These differences may contribute to their distinct substrate specificities. Together, these findings provide new insights into the functional organization of PUFA synthases, facilitating future rational optimization for biotechnological applications.

## 2 RESULTS

### 2.1 *Shewanella baltica* strain 6–42 *pfaB* is essential for PUFA synthesis

To assess the role of PfaB in gammaproteobacterial PUFA synthesis, we used the pDHA4 plasmid containing the *pfaA‐E* gene cluster from *M. marina* PUFA synthase, which enables DHA production in *Escherichia coli* (Giner‐Robles et al., 2018; Orikasa et al., 2009). In pDHA4, *pfaA‐D* are expressed under their native promoter, while *pfaE* expression is driven by a T7 promoter. Basal expression of the five genes was sufficient for DHA synthesis. *E. coli* BL21(DE3) transformed with pDHA4 produced DHA at 0.9% of total fatty acids, as determined by gas chromatography (GC; Table 1).

**TABLE 1:** PUFA production in *E. coli* BL21(DE3) expressing *Moritella marina* and *Shewanella baltica pfa* genes.

		Without IPTG			+ 0.1 M IPTG		
Vector carrying *M. marina pfa* cluster	Vector carrying *pfaB*	EPA (%)^a^	DHA (%)^a^	Unidentified FA^b^ (%)^a^	EPA (%)^a^	DHA (%)^a^	Unidentified FA^b^ (%)^a^
None	None	—	—	—	—	—	—
pDHA4	None	—	0.9	—	—	0.5	—
pDHA4	pET29c::*pfaBs*	— —	—	0.3 0.5	2.5	—	—
pDHA4	pET29c::*pfaBm*	—	1.0	—	—	—	—
pDHA4Δ*pfaB* ^c^	None	—	—	—	—	—	—
pDHA4Δ*pfaB*	pET29c::*pfaBs*	— —	—	0.7 0.6	2.3	—	—
pDHA4Δ*pfaB*	pET29c::*pfaBm*	—	0.5	—	—	—	—

*Note*: *pfaBm* refers to *pfaB* from *M. marina MP‐1*, which is a DHA‐producing cluster. *pfaBs* refers to *pfaB* from *S. baltica*, which is an EPA‐producing cluster.

^a^ Values correspond to the percentage of the peak area of the fatty acid in relation to the total area from all fatty acids.

^b^ Unidentified fatty acids: retention times did not correspond to any of the standards used.

^c^ pDHA4Δ*pfaB* refers to the plasmid pDHA4 with *pfaB* deleted. For details of vector constructions see Table S1.

Deletion of *pfaBm* in pDHA4 (pDHA4Δ*pfaBm*) abolished production, resulting in none or undetectable levels. Instead, a peak at 23.9 min was observed in the GC chromatogram under all conditions, including the strain harboring pDHA4Δ*pfaBm* (Figure S1d). This peak corresponds to a basal C18 fatty acid species derived from *E. coli*, accounting for 1% of total fatty acids and consistent with previous reports (Zheng et al., 2012). Complementation with pET29c::*pfaBm*, whose expression is controlled by a T7 promoter, partially restored synthesis (0.5%) (Figure S1e). In contrast, co‐expression of pET29c::*pfaBm* with pDHA4 maintained DHA production levels compared to the pDHA4 strain (Table 1; Figure S1f).

*Escherichia coli* strains harboring pDHA4 with pET29c::*pfaBm* function as complementation controls for the pDHA4Δ*pfaB* mutant. Supplying pET29c::*pfaBm* in trans confirmed that the loss of activity in the deletion strain is specifically due to the absence of *pfaBm*.

*Shewanella baltica* strain 6–42, isolated in Ny‐Ålesund (Norway), produces 8.8% of EPA relative to total fatty acids at 0°C (Peng et al., 2016). This strain harbors a *pfaA‐D* gene cluster similar to that of *M. marina*, and its PfaB (PfaBs) shares 28% sequence identity with PfaBm. Co‐expression of pDHA4Δ*pfaBm* or pDHA4 with pET29c::*pfaBs* led to additional peaks at 24.4 and 26.5 min (Figure S1e,f), consistent with the accumulation of longer chain (C18‐C20) intermediates. The presence of the 23.9 min peak remained unchanged, indicating that *pfaBs* expression enables chain elongation. Comparing these strains tests whether heterologous *pfaBs* can complement the deletion. Upon addition of 0.1 mM IPTG, distinct fatty acid profiles were observed. In pDHA4‐harboring strains, DHA production decreased from 0.9% to 0.5%. Moreover, DHA synthesis was abolished after pDHA4 co‐expression with pET29c::*pfaBm* or pDHA4Δ*pfaB* co‐expression with pET29c::*pfaBm*. In contrast, co‐expression of either pDHA4 or pDHA4Δ*pfaB* with pET29c::*pfaBs* yielded a peak at 32.76 min corresponding to EPA (Figure S1k,l), accounting for 2.5% and 2.3% of the total fatty acids, respectively (Table 1).

These results, consistent with previous reports (Orikasa et al., 2009), underline the key role of PfaB in determining the final PUFA product. They also suggest that the pathway can shift toward DHA or EPA depending on the PfaB variant.

### 2.2 PfaBm and PfaBs conserve acyltransferase activity

Gammaproteobacterial PfaB proteins typically contain KS′ and AT domains. InterPro analysis (Blum et al., 2025) identified a thiolase‐like fold within KS′ resembling a canonical KS domain, although it is shorter in PfaBs and therefore initially undetected.

Based on a representative selection of sequences, PfaB‐KS′ domains are presumably inactive, as they lack the canonical Cys‐His‐His catalytic residues. They share only ⁓18% identity with PfaA‐KS and PfaC‐KS, which contain the catalytic triad (Figure S2). In contrast, PfaA‐KS and PfaC‐KS themselves share ⁓33% identity. When comparing orthologous domains among DHA producers *M. marina* and *Colwellia* sp. MT41, sequence identity is 43% for PfaB‐KS′, 83% for PfaA‐KS, and 81% for PfaC‐KS. Among EPA producers *S. baltica* and *Photobacterium profundum*, the corresponding domains share 34%, 89%, and 78% identity, respectively. Cross‐comparison between DHA and EPA producers shows lower identity: <20% for PfaB‐KS′, <70% for PfaA‐KS, and <55% for PfaC‐KS (Figure S3).

PfaA‐MAT and PfaB‐AT domains share only ⁓20% identity, supporting the idea they could have distinct functions. As with previous analyses, these percentages are based on a representative selection of sequences. PfaB‐AT is more conserved among DHA producers (⁓70%) than in EPA producers (⁓50%), and cross‐group identity averages 30%. In contrast, PfaA‐MAT is more conserved (⁓50%). Between prokaryotic and eukaryotic PUFA synthases, average identity is ⁓20% for PfaB‐AT and ⁓35% for PfaA‐MAT (Figure S4). These differences suggest divergent substrate specificities, although the precise role of PfaB‐AT was unclear.

The biochemical activity of PfaBs and PfaBm was examined by in vitro radioactive binding and transfer assays. Both proteins were purified as monomers by size exclusion chromatography (SEC) (Figure S5). They were then incubated with radiolabeled substrates in the presence or absence of *M. marina* tandem holo‐ACP (5ACPm). Conversion from apo‐ACP to the holo form was confirmed by mass spectrometry (Figure S6), as previously described (Santín & Moncalián, 2018).

**FIGURE 2:**
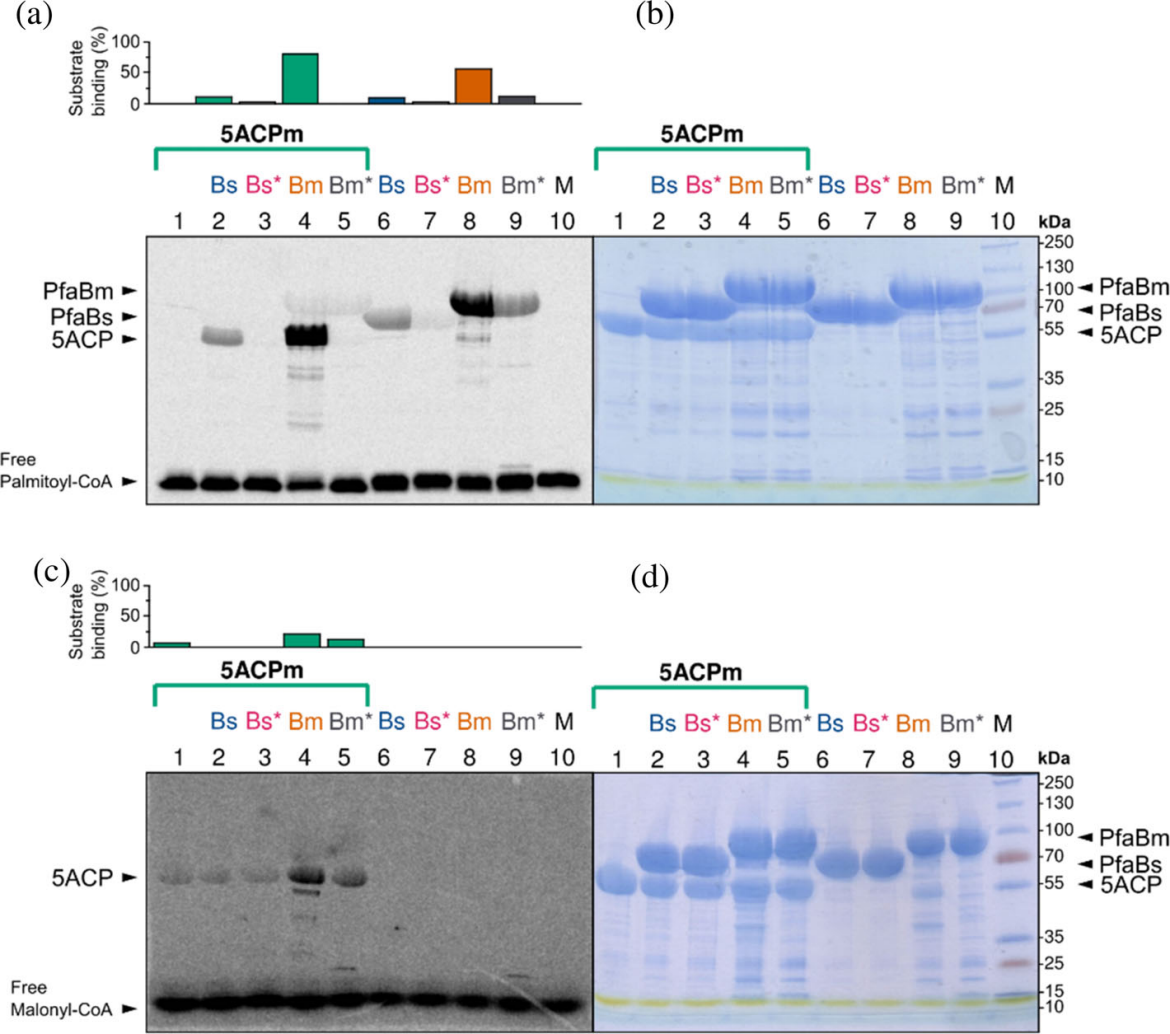
Ligand‐binding assays of 5ACPm with PfaB wild‐type and mutants. (a) 10% radio‐SDS‐PAGE showing 20 μM of PfaBm or PfaBs, incubated for 45 min in presence or absence of 20 μM of 5ACP with ^14^C‐palmitoyl‐CoA. (b) 10% SDS‐PAGE of purified PfaBm or PfaBs incubated in the presence or absence of 5ACP with ^14^C‐palmitoyl‐CoA, prior to autoradiography. (c) 10% radio‐SDS‐PAGE showing 20 μM of PfaBm or PfaBs, incubated for 45 min in presence or absence of 20 μM of 5ACP with ^14^C‐malonyl‐CoA. (d) 10% SDS‐PAGE of purified PfaBm or PfaBs incubated in the presence or absence of 5ACP with ^14^C‐malonyl‐CoA, prior to autoradiography The corresponding graphs quantify the amount of substrate covalently bound to each protein and are expressed as relative percentages. Band intensities were quantified by densitometric analysis. In panel c, relative percentages from lanes 2 to 5 were subtracted from that of lane 1, to exclude ACP self‐malonylation. Protein mapping positions are indicated by black arrows. 5ACPm, Acyl‐carrier protein; Bs, *S. baltica* PfaB; Bs* *S. baltica*, PfaB (S457A); Bm, *M. marina* PfaB; Bm*, *M. marina* PfaB (S608A); M, molecular mass marker.

PfaBm and PfaBs covalently bind ^14^C‐palmitoyl‐CoA (C16) (Figure 2a, lanes 6 and 8). Both proteins transferred the entirety of the previously bound palmitoyl‐CoA to 5ACPm (Figure 2a, lanes 2 and 4). Residual palmitoylated PfaBm remained detectable, whereas palmitoyl‐PfaBs is not detected, likely due to its lower substrate affinity.

Because ACPm domains perform self‐malonylation (Santín & Moncalián, 2018), the radioactive intensity values obtained in absence of PfaB‐AT were subtracted (Figure 2c, lane 1). Neither PfaBm nor PfaBs bound malonyl‐CoA (Figure 2c, lanes 6 and 8). Notably, PfaBm transferred the substrate upon incubation with 5ACPm, whereas PfaBs did not (Figure 2c, lanes 2 and 4). These results are consistent with previous radioactive assays, in which malonyl‐PfaBm was not detected and the acyltransfer reaction to ACP was less efficient than that of PfaA‐MAT (Santín & Moncalián, 2018). However, malonyl‐CoA transfer to ACP by PfaBs was not detected. Hayashi et al. (2020) showed that PfaB‐AT from *Schizochytrium* sp. (SsorfB) does not bind palmitoyl‐ACP, while *P. profundum* PfaB (EpaB) displays moderate binding. Other PfaB homologs exhibit hydrolase, but not acyltransferase activity (Almendáriz‐Palacios et al., 2021; Hayashi et al., 2020). In contrast, our results demonstrate that PfaBm and PfaBs bind palmitoyl‐CoA and transfer it to ACP, establishing their function as active AT enzymes in vitro.

### 2.3 PfaBm Y607 is crucial for binding and transfer

**FIGURE 3:**
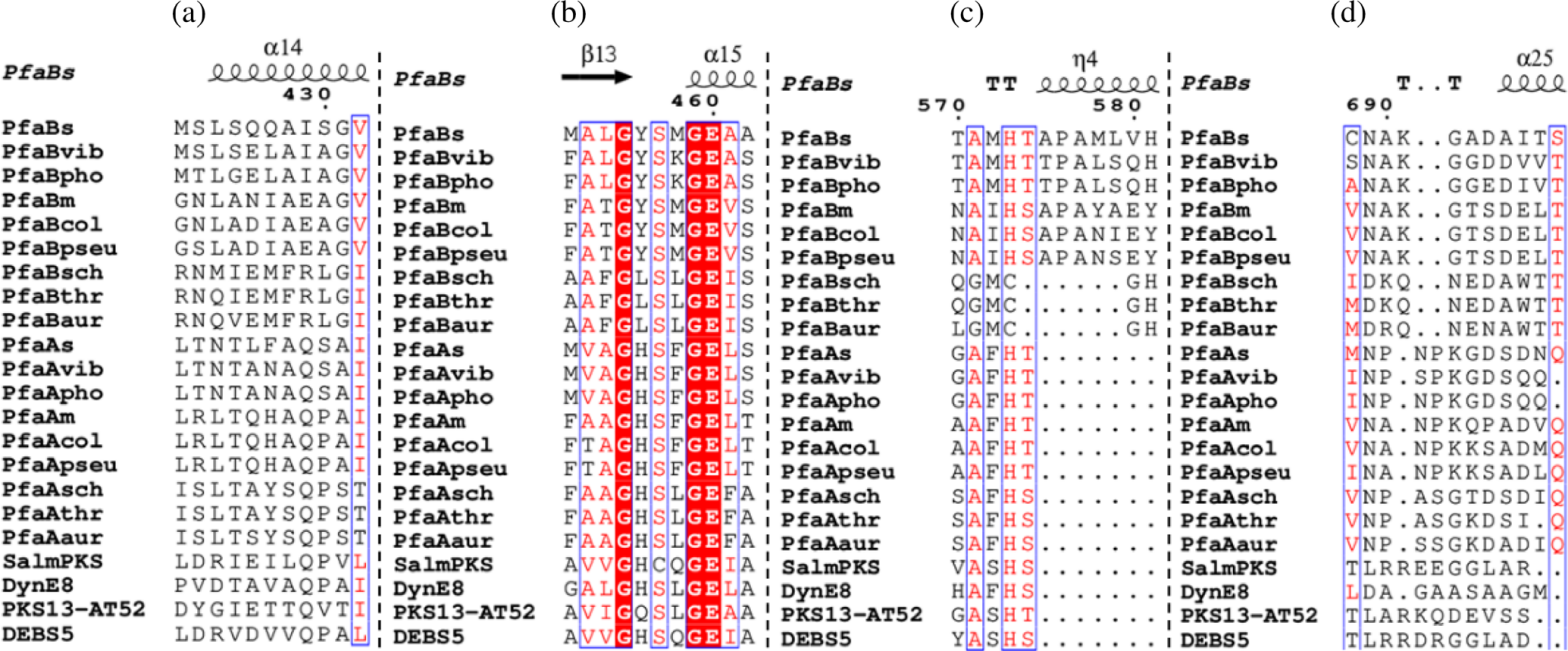
Structure‐based multiple sequence alignment of PfaBs‐AT. Residue numbering corresponds to PfaBs‐AT. Identical residues present in all sequences are shown as white letters on a red background, while conserved residues are indicated in red. Secondary structure elements of PfaBs‐AT (arrows for β‐strands, and coils for α‐helices) are indicated at the top of the alignment. (a) Motif I. (b) Motif II. (c) Motif III. (d) Motif IV. UniProt accession numbers of the proteins included in the alignment are as follows (NCBI entries are indicated where applicable): PfaBs, *S. baltica* PfaB (NCBI: OZ287685); PfaBvib, *Vibrio splendidus* (A0AB35N5F5); PfaBpho, *Photobacterium profundum* (Q93CG7); PfaBm, *M. marina* (A0A5J6WK78); PfaBcol, *Colwellia* sp. MT41 (A0A0S2JES7); PfaBpseu, *Pseudoalteromonas denitrificans* (A0A1I1RM50); PfaBsch, *Schizochytrium* sp. (Q94FB7); PfaBthr, *Thraustochytrium* sp. (A0A1B3PEI8); PfaBaur, *Auriantiochytrium* sp. (A0A7H0U711); PfaAs, *S. baltica* (NCBI: OZ346655); PfaAvib, *V. toranzoniae* (A0A125P5E0); PfaApho, *P. profundum* (Q93CG8); PfaAm, *M. marina* (A0A5J6WHR2); PfaAcol, *C*. sp. MT41 (A0A0S2JKT5); PfaApseu, *P. denitrificans* (A0A1I1REG2); PfaAsch, *Schizochytrium* sp. (Q94FB8); PfaAthr, *Thraustochytrium* sp. (A0A1B3PEI6); PfaAaur, *Auriantiochytrium* sp. (A0A7H0ZVK4); SalmPKS, *Streptomyces albus* (H6D573); DynE8, *Micromonospora chersina* (Q84HI8); PKS13‐AT52, *Mycobacterium tuberculosis* (O53579); DEBS5, *Saccharopolyspora erythraea* (Q03133). The alignment was plotted with ENDscript.

Four conserved motifs are found in AT domains (Park et al., 2014). Motif I (VDVVQ), located ⁓30 residues upstream of the catalytic serine, influences substrate specificity. Its variants include V[DE]VVQ in methylmalonyl‐AT (MMAT) and xx[AT][QE] in MAT (Liew et al., 2012; Yadav et al., 2003). In gammaproteobacterial PfaB the motif corresponds to [SA]xxA[IE] and IEMFR in thraustochytrid PfaB (Figure 3a). Motif II (Gx**S**xG), fully conserved in PfaB, contains the catalytic serine responsible for nucleophilic attack on the acyl‐CoA thioester (Keatinge‐Clay et al., 2003) (Figure 3b). Motif III (xAx**H**) includes the catalytic histidine, which acts as a general base. The motif xAFH is MAT‐specific, while [YVW]ASH characterizes MMAT (Smith & Tsai, 2007). In prokaryotic PfaB, the motif is [NT]A[IM]H[ST], whereas eukaryotic PfaB features MCGH (Figure 3c). Motif IV also contributes to substrate specificity in modular PKS‐AT. It is located at the C‐terminus in a variable region (Lau et al., 1999). This corresponds to residues 663–713 in PfaBs and 807–861 in PfaBm. The consensus sequence NAKGx[DES] is conserved in bacterial PfaB, while eukaryotic PfaB shows D[KR]QNED. In PfaA‐MAT, the motif is N[AP]NPK[GKQ] (Figure 3d).

To evaluate the functional relevance of these residues we performed site‐directed mutagenesis. Substitution of the catalytic serine with alanine, PfaBm* (S608A) and PfaBs* (S457A), resulted in a reduced ability to bind ^14^C‐palmitoyl‐CoA (Figure 2a, lanes 7 and 9). A band of palmitoyl‐PfaBm* is observed (Figure 2a, lane 9), but not for PfaBs* (Figure 2a, lane 7). This is likely given the lower affinity of the wild‐type PfaBs for palmitoyl‐CoA. In the presence of ACP, AT activity was lost (Figure 2a, lane 5). In addition, palmitoyl‐PfaBm was barely detectable, indicating that the active serine is essential for the acyl‐enzyme intermediate stability (Figure 2a, lane 9).

**FIGURE 4:**
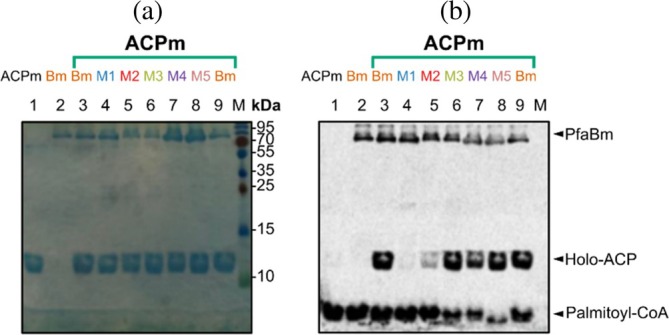
Binding assays of PfaBm mutants and ACPm. (a) 15% SDS‐PAGE of purified PfaBm mutants and ACPm incubated with ^14^C‐palmitoyl‐CoA, prior to autoradiography. (b) 15% radio‐SDS‐PAGE showing 20 μM of PfaBm mutants and 20 μM of ACPm incubated with ^14^C‐palmitoyl‐CoA. ACPm (Acyl‐carrier protein); Bm, *M. marina* PfaB wild type; M1, PfaBm (Y607H); M2, PfaBm (Y607L); M3, PfaBm (S608C); M4, PfaBm (M609F); M5, PfaBm (I728F); M, molecular mass marker.

PfaBm Y607 from motif II is conserved in bacterial PfaB proteins. In eukaryotic PfaB this residue is replaced by leucine, and in PfaA‐MAT by histidine. To determine its role, mutants M1 (Y607H) and M2 (Y607L) were constructed. AT activity assays were performed using one PfaA‐ACPm domain (PfaA residues 1255–1348). These experiments suggest that Y607 is crucial for catalysis, as both M1 (Figure 4b, lane 4) and M2 (Figure 4b, lane 5) have reduced activity in vitro. Mutant M3 (S608C) retained the ability to transfer ^14^C‐palmitoyl‐CoA (Figure 4b, lane 6). This is likely given the similar side‐chain size of serine and cysteine, and the comparable nucleophilic potential of their hydroxyl and thiol groups. This observation is consistent with previous studies on the DSZS PKS‐MAT, where self‐acylation and transfer to ACP were detected, although with a 200‐fold decrease in K_cat_/K_M_ (Wong et al., 2011).

Mutant M4 (M609F) changed a methionine present in the active site of certain bacterial PfaB to phenylalanine, which is conserved in bacterial PfaA‐MAT. Similarly, mutant M5 (I728F) substituted the isoleucine adjacent to the catalytic H729 for the conserved phenylalanine in PfaA‐MAT (Figure 3c). Both M4 and M5 retain AT activity, indicating that these residues are not essential for catalysis (Figure 4b, lanes 7 and 8).

Together, these results show that the catalytic serine is essential for AT activity, and that the conserved Y607 in PfaBm is critical for binding and transfer of palmitoyl‐CoA.

### 2.4 PfaB transfers long‐chain acyl substrates

To determine PfaBm and PfaBs substrate specificity and assess their activity, we performed liquid chromatography‐mass spectrometry (LC–MS) analyses. Each protein was incubated with holo PfaA‐ACPm and various acyl‐CoA substrates.

**FIGURE 5:**
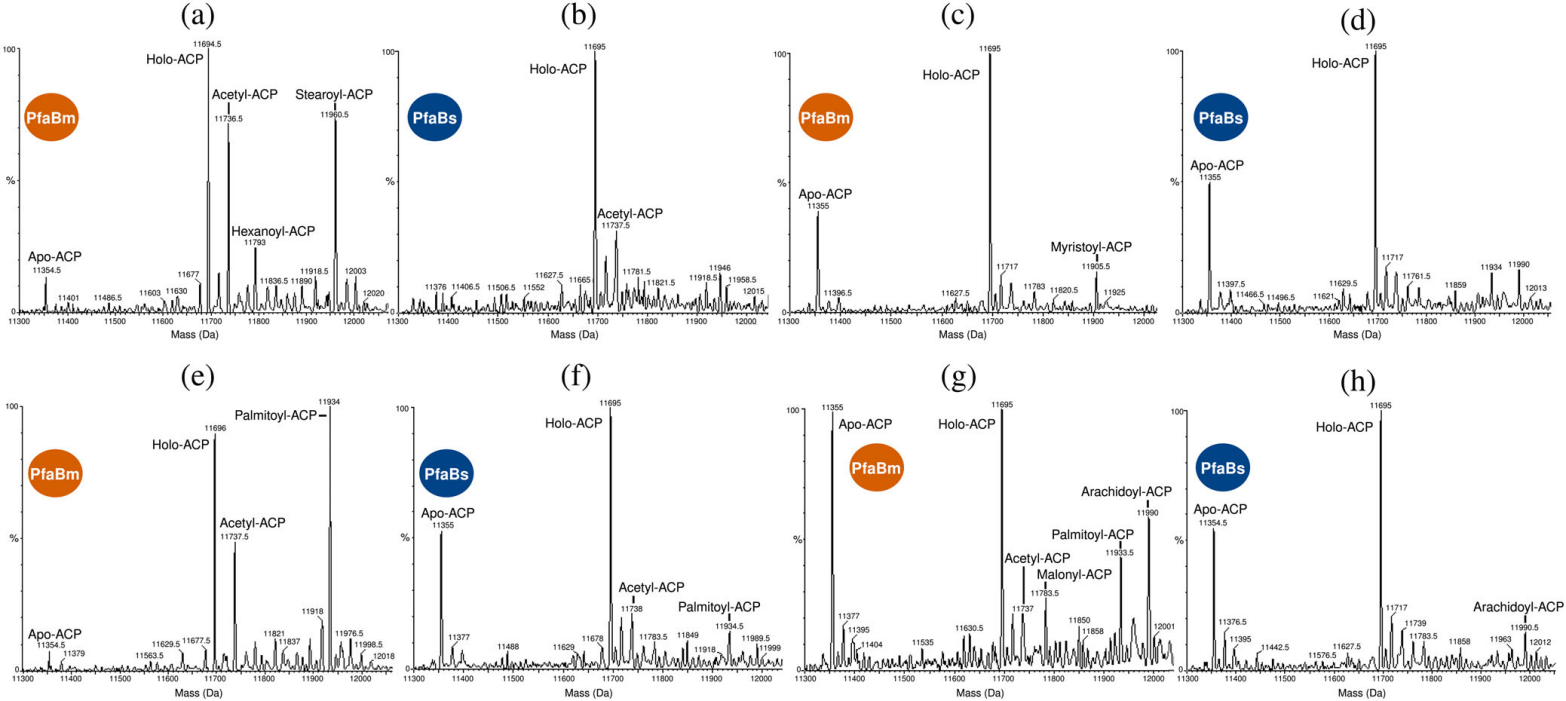
Mass spectrometric analysis of reactions catalyzed by PfaBm and PfaBs after incubation with PfaA‐ACPm and different substrates. (a) PfaBm with hexanoyl‐CoA. (b) PfaBs with hexanoyl‐CoA. (c) PfaBm with myristoyl‐CoA. (d) PfaBs with myristoyl‐CoA. (e) PfaBm with palmitoyl‐CoA. (f) PfaBs with palmitoyl‐CoA. (g) PfaBm with arachidoyl‐CoA. (h) PfaBs with arachidoyl‐CoA. Forty micrograms of protein were analyzed. Spectra were manually acquired over an m/z range of 11,300–12,500, and deconvolution was performed using default parameters.

PfaBm inefficiently transfers hexanoyl‐CoA (C6) to PfaA‐ACPm, indicating low activity with short‐chain substrates. Additionally, acetyl‐ACP (C2) and stearoyl‐ACP (C18) were also detected in the spectra, likely reflecting purification contaminants (Figure 5a). In contrast, PfaBs showed no detectable activity with hexanoyl‐CoA (Figure 5b). PfaBm weakly transfers myristoyl‐CoA (C14) to PfaA‐ACPm (Figure 5c), whereas PfaBs do not transfer this ligand (Figure 5d).

Both PfaBm and PfaBs transfer palmitoyl‐CoA to PfaA‐ACPm, although the reaction is less efficient for PfaBs (Figure 5e,f). PfaBm additionally transfers arachidoyl‐CoA (C20) to PfaA‐ACPm (Figure 5g), while PfaBs transfer this substrate with lower efficiency (Figure 5h).

These results, consistent with our radioactive assay observations, further confirm that both PfaBm and PfaBs preferentially transfer long‐chain acyl substrates.

### 2.5 PfaBs overall structure

**FIGURE 6:**
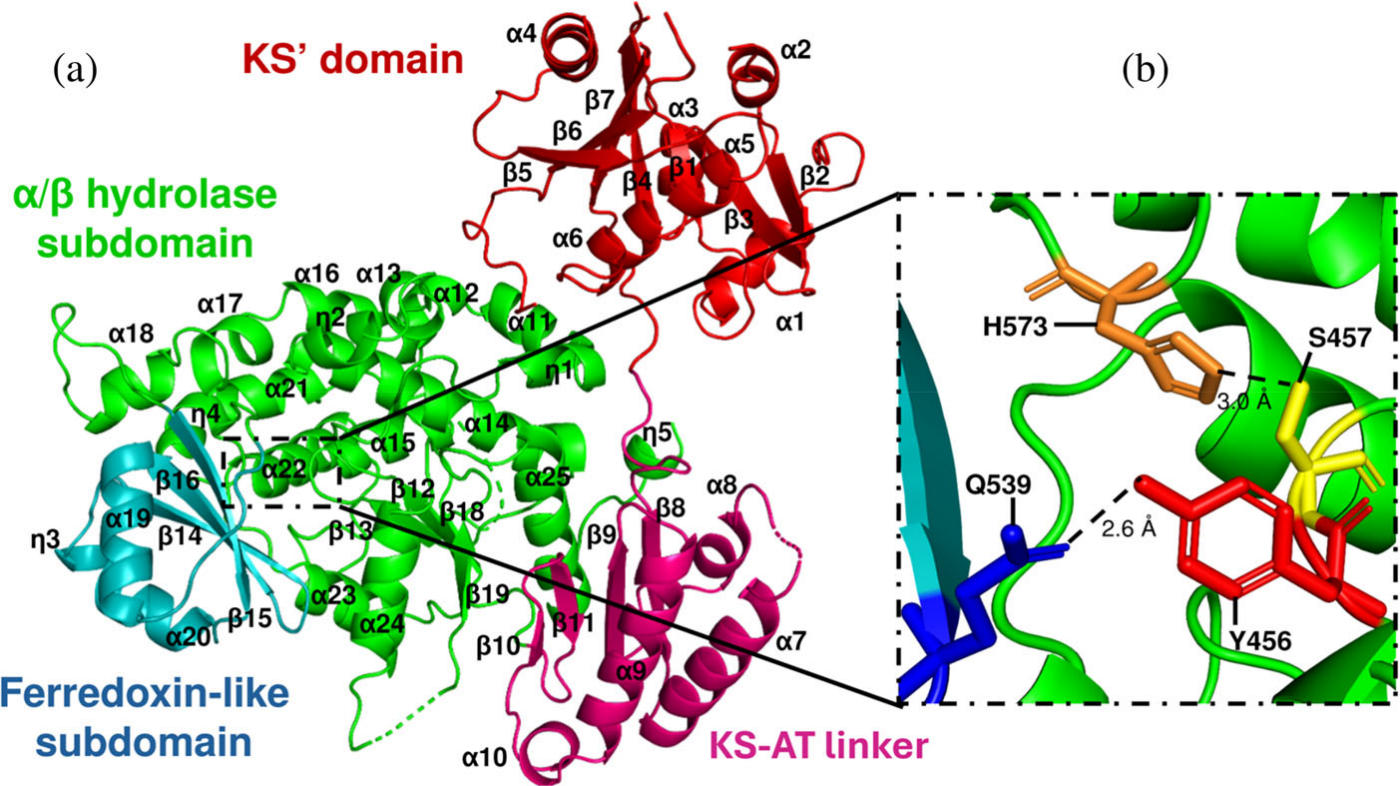
Overall structure of PfaBs. (a) PfaBs is contains a KS′ domain (red), a KS′‐AT linker (magenta) and an AT domain (cartoon representation), which is composed of a ferredoxin‐like subdomain (cyan), and α/β hydrolase subdomain (green). (b) PfaBs active site. Catalytic S457 (yellow sticks) and H573 (orange sticks) residues are shown. Y456 (red sticks) and Q539 (blue sticks) form a conserved hydrogen bond. Dashed lines represent the distance between residues.

The PfaBs crystal structure (PDB ID: 9RY8) was solved at 2.9 Å by molecular replacement using an AlphaFold3 model (Abramson et al., 2024) (Table 2), providing the first detailed view of this enzyme within PUFA megasynthases. PfaBs adopts a two‐domain architecture composed of an N‐terminal KS′ domain (residues 1–255), a linker (256–361) and a C‐terminal AT domain (362–720) (Figure 6a).

**TABLE 2:** Data collection and refinement statistics of the PfaBs crystal structure (molecular replacement).

PfaBs (PDB 9RY8)	
Data collection	
Space group	P4_1_2_1_2
Unit cell, α = β = γ = 90	81.870, 81.870, 210.983
Wavelength (Å)	0.97929
Resolution range (Å)	64.7–2.9
Reflections total/unique	30,349 / 16,672
% completeness (last shell)	100 (99.8)
Multiplicity (last shell)	1.8 (1.9)
Average *I/σ* (last shell)	24.4 (7.5)
Rmerge (last shell)	0.1 (0.537)
CC1/2 (last shell)	1.000 (0.814)
Refinement	
R_work_/R_free_	0.2305 / 0.3195
Protein atoms	4667
Wilson B‐factor (Å^2^)	77.4
OWAB per residue (min, median, 95th, max; Å^2^)	42, 68, 107, 131
RSRZ outliers (n, %)	5 (0%)
Root mean square deviations	
Bond lengths (Å)	0.008
Bond angles (°)	0.97
Ramachandran plot	
Favored	94.2
Allowed	5.8
Outlier	0.0

The KS′ domain lacks the canonical catalytic triad and shows weak electron density in several regions, suggesting a high degree of flexibility. This observation is supported by refinement statistics: the highest occupancy‐weighted average B‐factor (OWAB) values correspond to KS′ residues. On the other hand, the OWAB values for the AT domain are closer to the median, suggesting greater stability (Table 2). Within the resolved KS′ region, six α‐helices and seven β‐sheets were identified. Certain segments (e.g., residues A128–N142, T230–N241, K229) show poorly defined or absent density, indicating local disorder (Figure S7a). In contrast, the AT domain displays a well‐defined α/β hydrolase and ferredoxin‐like subdomains, characteristic of acyltransferases (Figure 6a).

Despite sharing only ⁓15% sequence identity with other PKS‐AT enzymes, such as *Streptomyces albus* salinomycin mPKS‐AT module 8 (PDB 6IYR; RMSD 5.71) (Zhang et al., 2019), or *Mycobacterium tuberculosis* PKS13‐AT52 domain (PDB 3TZW; RMSD 4.19) (Bergeret et al., 2012), PfaBs preserves the characteristic overall fold of this enzyme family. The α/β hydrolase subdomain (residues 362–511 and 570–725) contains 17 α‐helices, four η‐helices, and nine β‐sheets, while 11–13 α‐helices are often found in PKS‐AT and 15 for PfaBm AlphaFold3 prediction (RMSD 1.31). The ferredoxin‐like subdomain (residues 512–569) includes two α‐helices, one η‐helix, and three anti‐parallel β‐sheets (Figure 6a). In PKS‐AT and PfaAs‐MAT AlphaFold3 models (residues 498–960; RMSD 5.09), this subdomain contains an extra β‐sheet. The KS′‐AT linker forms an α/β fold, with four α‐helices and four anti‐parallel β‐sheets, as described for *Micromonospora chersina* DynE8‐MAT (PDB 4AMO; RMSD 12.20) (Liew et al., 2012) and AlphaFold3 predictions for PfaAs‐MAT and PfaBm (RMSD 1.31). However, in *Saccharopolyspora erythraea* DEBS module 5 (PDB 2HG4; RMSD 14.05) (Tang et al., 2007), or PKS13‐AT52 domain (PDB 3TZW; RMSD 4.19) (Bergeret et al., 2012), this linker is formed by three β‐sheets.

These structural features confirm that PfaBs retain the architecture of AT domains. Notably, residues W501 and W511 are fully conserved in PfaB proteins (Figure S8). The stacking interaction between these residues stabilizes a negatively charged loop absent in other AT domains, potentially mediating interactions with other components of the PUFA synthase complex (Figure S9).

### 2.6 PfaBs active site

The active site of PfaBs, located in the AT domain, is supported by well‐defined electron density at 2.9 Å resolution, including the catalytic serine and surrounding residues (Figure S7b). Consistent with other AT domains, the PfaBs active site lies in a deep cleft between the α/β hydrolase and ferredoxin‐like subdomains. Motif II, Gx**S**xG (GY**S**MG^459^ in PfaBs), contains the catalytic S457, located in a turn between β13 and α15. In PKS‐MAT, FAS‐MAT, and PfaA‐MAT, this motif is GH**S**xG (Figure 3b); where the conserved histidine stabilizes the malonyl‐enzyme intermediate preventing hydrolysis (Poust et al., 2014). In bacterial PfaB‐AT, this histidine is replaced by a tyrosine. The PfaBs crystal structure shows a Y456–Q539 hydrogen bond (Figure 6b). In DynE8‐MAT (Liew et al., 2012), the corresponding histidine adopts a similar orientation. It forms hydrogen bonds with a conserved asparagine in the ferredoxin‐like subdomain. Via a water bridge, this histidine also interacts with the MAT ester carbonyl oxygen, stabilizing the acyl‐enzyme intermediate. The conserved asparagine is present in DHA‐type PfaB, but replaced by Q539 in PfaBs, as other EPA‐type PfaB proteins (Figure S8).

Motif III (xAx**H**) contains the catalytic histidine that activates the catalytic serine (Keatinge‐Clay et al., 2003; Liew et al., 2012). (Smith & Tsai, 2007) In PfaBs, this corresponds to TAM**H** ^573^ forming a Ser‐His dyad (3 Å distance, Figure 6b). Conservation and spatial arrangement suggest a mechanism similar to other MATs, although motif variability may influence substrate specificity.

MAT and MMAT domains contain conserved glutamine and arginine residues. These are essential for carboxylate transfer and malonyl‐CoA specificity. They form a stabilizing salt bridge with the carboxyl group from the ligand. In PfaB proteins, this arginine is absent (replaced by N482 in PfaBs; Figure S8), suggesting a different substrate interaction. Similarly, the conserved glutamine from motif I (VDVVV**Q**) is not present in bacterial PfaB proteins; it is replaced in PfaBs by I429, a residue found in other EPA‐type PfaB. On the other hand, DHA‐type PfaB proteins contain a glutamate in this position.

The oxyanion hole in AT domains is constituted by two residues and stabilizes the carbonyl oxygen during the transition state. In *E. coli* FabD‐MAT, Q11 and L93 (next to catalytic S92) fulfill this role (Serre et al., 1995). Q11 is part of a conserved PG**Q**xQ motif found in FAS, PKS, and PfaA‐MAT (Keatinge‐Clay et al., 2003; Liew et al., 2012; Serre et al., 1995). In PfaBs it is replaced by PG**V** ^374^GT. The first residue forming the oxyanion hole is valine in EPA‐type PfaB, isoleucine in DHA‐type PfaB, and glycine in eukaryotic PfaB‐AT (Figure S8). The second residue, adjacent to the catalytic serine, corresponds to Leu/Ile/Val in MAT domains (Park et al., 2014), or Phe in PfaA‐MAT and other AT domains (e.g., ZmaA‐AT). PfaBs contain M458 and PfaBm M609 in this position (Figure 3b). The presence of bulky residues at this site, such as methionine or phenylalanine, may restrict binding of substrates like methylmalonyl‐CoA. This could contribute to differences in substrate specificity (Tang et al., 2006).

Motif IV, located in the C‐terminal region (⁓30 residues), also influences substrate specificity (Lau et al., 1999). In PKS and FAS‐AT structures, an α‐helix in this region contributes to the substrate channel exit, a feature conserved in PfaBs crystal structure and PfaA‐MAT AlphaFold3 models.

The detailed analysis of the PfaBs active site reveals conserved catalytic residues arranged in a Ser‐His catalytic dyad, alongside distinct variations in key substrate‐binding motifs compared to other AT domains.

### 2.7 Acyl‐ACP substrate docking

**FIGURE 7:**
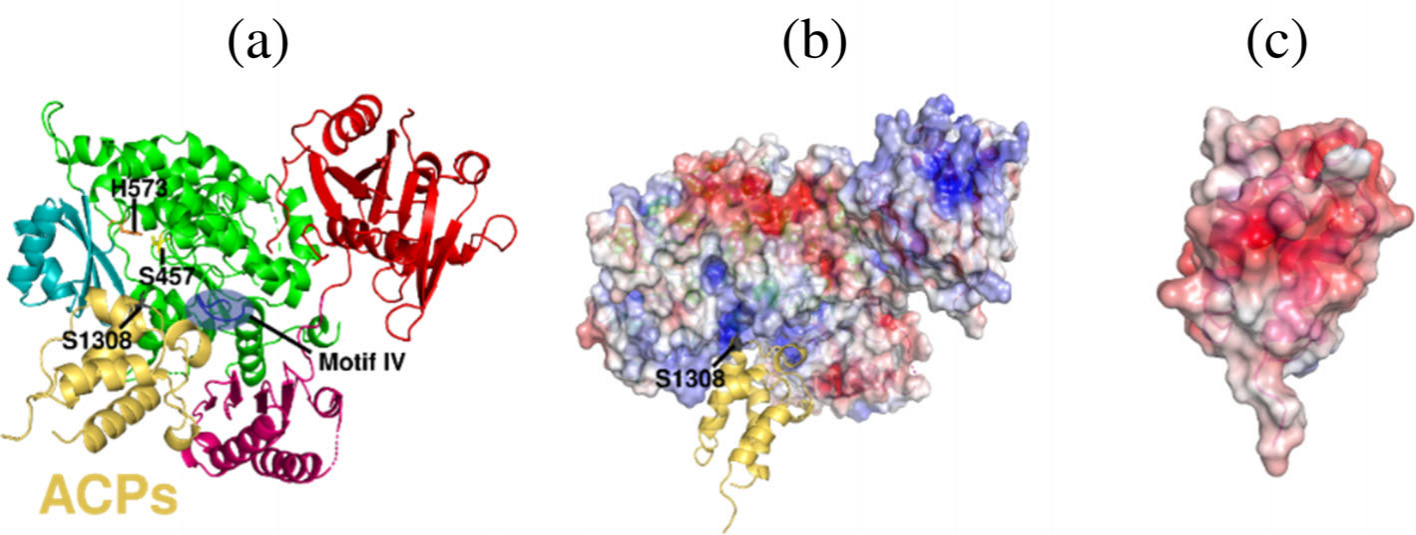
AlphaFold3 ACPs docking prediction and alignment with the PfaBs crystal structure. (a) ACPs (yellow‐orange cartoon, active S1308 shown as gray sticks), binds PfaBs in a pocket delimited by the KS‐AT linker, and the ferredoxin‐like subdomain via motif IV (blue). (b) Electrostatic surface map of PfaBs, generated with the adaptive Poisson‐Boltzmann solver (APBS) in PyMOL. Colors range from blue (positive) through white to red (negative). The interacting region with ACPs shows a positive electrostatic potential (blue). (c) Electrostatic surface potential representation of ACPs. The total accessible surface is highly electronegative (red).

Molecular docking of apo‐ACP (residues 1269–1353 from *S. baltica* PfaA) and PfaBs was performed with AlphaFold3 (Abramson et al., 2024). In the resulting models, ACP binds within a pocket formed by β‐strands of the ferredoxin‐like subdomain together with α9 and β11 from the KS′‐AT linker. Additional contacts involve the loop defined by the sequence NAKGAD^694^ (motif IV). Moreover, the active S1308 from PfaAs‐ACP is predicted to be oriented toward the PfaBs‐AT active site (Figure 7a). The interaction is largely electrostatic, with the electronegative ACP surface complementing the electropositive PfaBs pocket (Figure 7b,c). A comparable binding mode is observed in the salinomycin ACP‐mPKS‐AT module 9 complex (PDB 7VRS; RMSD 11.05) (Feng et al., 2022).

**FIGURE 8:**
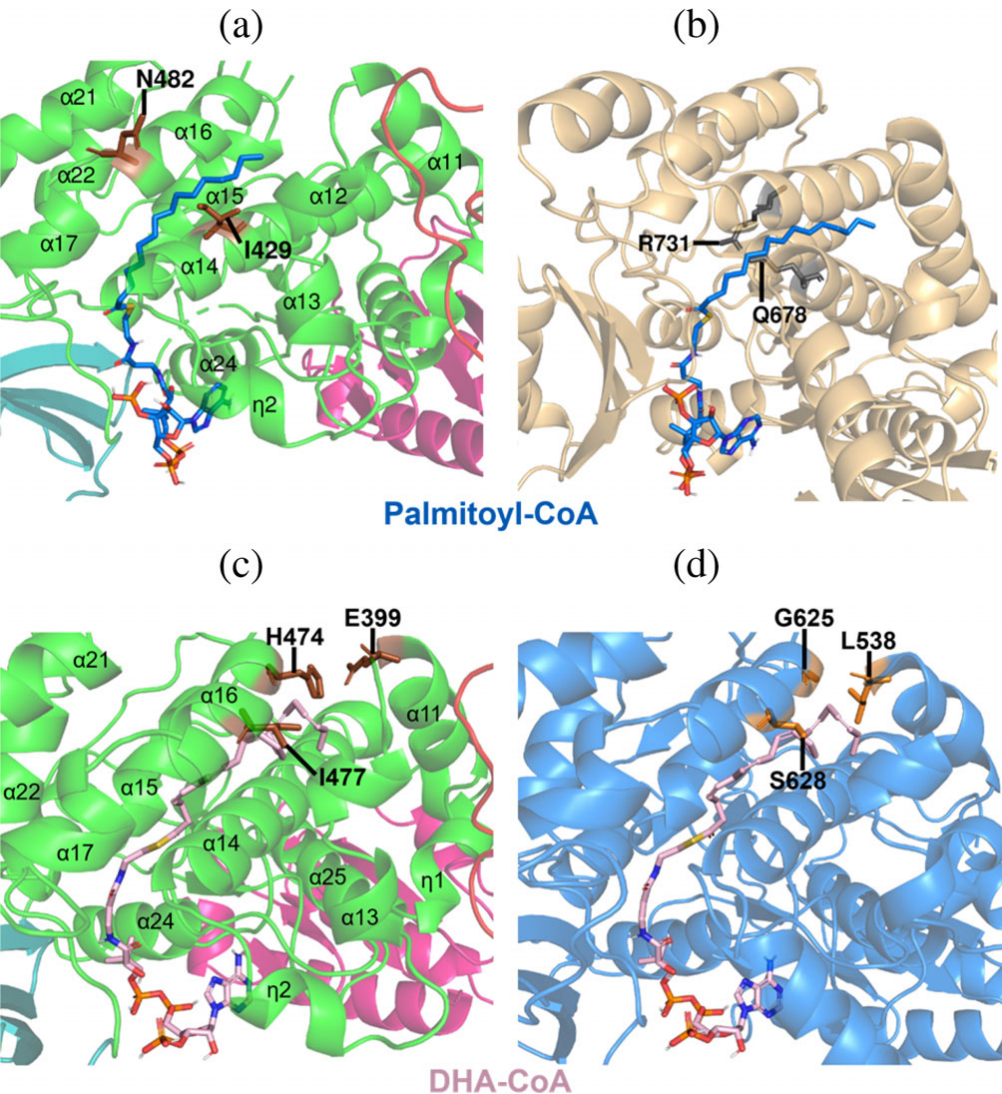
Acyl‐CoA predicted docking models of PfaB proteins. Docking was performed with SwissDock (AutoDock Vina). The search space was centered on the catalytic S457 of PfaBs for palmitoyl‐CoA and on S608 of the PfaBm AlphaFold3 model for DHA‐CoA. (a) Palmitoyl‐CoA (blue sticks) binds PfaBs in a pocket formed by helices α14‐α17. Residue I429 (brown sticks) from helix α14 contributes to the accommodation of long chain acyl substrates. (b) Palmitoyl‐CoA docking after structural alignment of PfaBs with the AlphaFold3 prediction of PfaA‐MAT (yellow–orange ribbon). The conserved Q678 and R731 (gray sticks) sterically clash with the substrate. (c) DHA‐CoA (pink sticks) docking in PfaBs following structural alignment with PfaBm. Residues E399 from helix α11, and H474 with I477 are positioned to form a lid that may hinder binding of long‐chain acyl substrates such as DHA‐CoA. (d) DHA‐CoA docking in PfaBm (blue ribbon representation). Residue L538 is closer to the ligand than its PfaBs counterpart E399 and may function as a lid. Additionally, residues G625 and S628 provide improved accommodation for DHA‐CoA.

Docking of PfaBs with acyl‐CoA using SwissDock positions the CoA moiety near the ACP‐interacting surface (Figure 8a). The carboxyl group is oriented toward the active site, while the acyl chain inserts into a negatively charged lipophilic tunnel delimited by helices α14–α17. Motif I (LSQQAI^429^) in α14 may contribute to the accommodation of long acyl chains. In contrast to the conserved Q678 and R731 in PfaAs‐MAT, residues I429 and N482 in PfaBs do not sterically clash with palmitoyl‐CoA (Figure 8b).

DHA‐CoA docks in a similar orientation, with potential contributions from helix α11 (Figure 8c), where E399 in PfaBs corresponds to L538 in PfaBm. In the models, L538 could act as a lid, approaching DHA (3.4 Å from its Cβ to DHA‐CoA C2) (Figure 8d). Multiple‐sequence alignment shows that this position is a conserved Glu in EPA‐type PfaB, Leu in DHA‐type PfaB, and Lys in thraustochytrids (Figure S8).

Helices α15‐α17 shape the predicted substrate pocket. Helix α15 in PfaBs differs in both length and sequence from PfaBm. A conserved motif, M[WY]A[SA]L^467^, located in α15 from PfaBs, is characteristic of gammaproteobacterial PfaB proteins: MYAAL in DHA‐type PfaB, and MWASL in EPA‐type PfaB. Eukaryotic PfaB proteins contain MxFxF at the corresponding position (Figure S8).

Helix α16 contains a second conserved motif, P[**HG**][AL][ML][**IS**]^477^, which differs between DHA‐ and EPA‐type PfaB and may influence substrate accommodation. DHA‐type PfaB exhibits the sequence P**G**LM**S**, whereas EPA‐type PfaB contains P**H**A[ML]**I**. Eukaryotic PfaB proteins instead show S[DE]xLT at this position (Figure S8). According to the docking models, H474 and I477 in PfaBs sterically clash with DHA‐CoA (Figure 8c). In contrast, the corresponding conserved residues G625 and S628 in PfaBm do not interfere with this DHA‐CoA docking, suggesting improved accommodation of longer acyl chains (Figure 8d). The bulkier residues in PfaBs may instead form a lid better suited for the shorter EPA acyl chain.

Finally, helices η2 and α14 differ between PfaBs and PfaBm. Helix η2 extends from 1.5 turns in PfaBs to 3.5 in PfaBm. The loop connecting η2 to α14 is one residue shorter in PfaBm (Figure 8d). This shortening may enlarge the tunnel exit, potentially facilitating accommodation of longer acyl chains.

Overall, these docking predictions suggest that ACP and acyl‐CoA substrates interact with PfaBs through conserved structural motifs and unique helices that shape the proposed binding pocket. The observed structural differences between PfaBs and PfaBm provide a framework for understanding chain‐length preferences. However, experimental validation is required to confirm these hypotheses.

## 3 DISCUSSION

In FAS, PKS and Pfa systems, AT domains load starter or extender units onto ACPs during chain elongation. While AT structures and catalytic mechanisms have been well characterized for FASs and PKSs (Liew et al., 2012; Serre et al., 1995; Zhang et al., 2019), here we report the first crystal structure of an AT domain from a Pfa system. We also integrate structural, biochemical, and in vivo data to assess the role of PfaB in PUFA biosynthesis.

Crystallography and SEC analyses indicate that PfaBs is a monomer (Figure S5). The protein comprises an N‐terminal KS′ pseudo‐domain and a canonical AT domain. The KS′ region is poorly conserved and orthologs are not present in eukaryotic PUFA synthases. It also lacks the conserved catalytic Ser‐His‐His triad and ACP/PPT‐binding motifs (Santín et al., 2020) (Figure S2), suggesting a non‐catalytic role. Instead, KS′ likely serves as a structural scaffold and may contribute to ACP positioning, consistent with the role of KS‐AT linkers in modular PKS‐MAT systems (Bergeret et al., 2012; Feng et al., 2022; Liew et al., 2012).

### 3.1 PfaB is a long‐chain acyltransferase

In vitro LC–MS and radioactive assays demonstrate that both PfaBs and PfaBm bind long‐chain acyl‐CoA substrates (>C16) and catalyze acyl transfer to ACP. PfaBm exhibited higher affinity for ^14^C‐palmitoyl‐CoA, while both proteins showed comparable transferase efficiency (Figure 2a, lanes 2 and 4). Rapid acyl transfer by PfaBm results in a residual palmitoylated enzyme band in the presence of the tandem 5ACPm construct, largely absent in PfaBs, consistent with its lower substrate affinity. However, when we used a single ACPm domain, we could observe a band of palmitoyl‐PfaBm (Figure 4, lanes 3–8). This suggests that the 5ACPm construct creates an excess of acyl acceptors relative to PfaB.

Previous studies showed that PfaAm‐MAT transfers malonyl‐CoA to ACP more efficiently than PfaBm (Santín & Moncalián, 2018). PfaBs does not detectably transfer malonyl‐CoA, or such transfer occurs below the detection limit (Figure 2c, lanes 2 and 4). These observations support functional specialization: PfaA‐MAT catalyzes extender‐unit loading onto ACP, while PfaB selectively processes long‐chain acyl intermediates.

Earlier reports attributed hydrolase activity to selected PfaB homologs, preferentially acting on long‐chain acyl substrates (Almendáriz‐Palacios et al., 2021; Hayashi et al., 2020). This substrate specificity is fully compatible with our observations, as both PfaBm and PfaBs selectively process long‐chain (>C16) acyl substrates, while shorter chains are poorly transferred. In our in vitro assays, PfaBm displayed higher activity than PfaBs and appeared more permissive with the long‐chain saturated acyl‐CoA substrates tested. These differences likely reflect distinct behaviors of the two enzymes under the assay conditions and with commercially available acyl‐CoA substrates rather than intrinsic substrate preferences within the native PUFA synthase system.

### 3.2 Catalytic serine stabilizes a transient acyl‐enzyme intermediate

Mutation of the catalytic serine (PfaBm* S608A; PfaBs* S457A) abolishes acyltransferase activity in vitro (Figure 2a, lanes 3 and 5), confirming its essential role in productive acyl transfer. The PfaBm* and PfaBs* mutants retain weak palmitoyl‐CoA binding (Figure 2a, lanes 7 and 9), probably indicating non‐specific unproductive binding, as further acyltransferase activity was completely impaired in this mutant.

Palmitoylation of PfaBm* is strongly reduced in the presence of ACP (Figure 2a, lane 5), suggesting that ACP destabilizes the acyl‐enzyme intermediate when the active serine is absent. Under these conditions, the acyl group is rapidly lost, preventing accumulation of detectable palmitoylated PfaBm*. These observations support the existence of a transient acyl–enzyme intermediate, whose stabilization depends on the catalytic serine upon ACP engagement. Although in vivo complementation with these mutants was not performed, the deletion pDHA4Δ*pfaBm* phenotype combined with the loss of activity observed in vitro supports that the enzymatic function of PfaB is essential for proper PUFA synthesis (Table 1).

### 3.3 Structural features distinguish PfaB from canonical hydrolases

Because the motif II (Gx**S**xG) and the α/β hydrolase fold are shared among acyltransferases, esterases, and other hydrolases, promiscuous activity was observed. However, the structural determinants that distinguish acyltransferase from hydrolytic function remain unclear (Müller et al., 2021). The PfaBs crystal structure reveals several features that clearly distinguish it from canonical hydrolases.

Structural alignment of PfaBs with representative hydrolases, such as *Pseudomonas aeruginosa* lipase (PDB 1EX9) shows substantial divergence (RMSD 17.31 Å). Notably, PfaBs lack the canonical Ser‐His‐Asp catalytic triad characteristic of esterases and lipases. Instead, its active site consists of a S457‐H573 dyad, embedded within a hydrogen bond network involving the conserved Y456 and Q539 (Figure S8). This organization is more closely related to PKS‐MAT domains, which are specialized to stabilize acyl–enzyme intermediates and suppress water‐mediated hydrolysis.

In PKS‐MAT domains, a histidine adjacent to the catalytic serine plays a key role in stabilizing the malonyl‐enzyme intermediate (Poust et al., 2014). In contrast, PfaB proteins conserve tyrosine at this position (Y607 in PfaBm, Y456 in PfaBs). Mutagenesis confirmed that this tyrosine is essential for PfaBm activity. Moreover, it is not interchangeable with histidine or leucine found in MATs and certain eukaryotic PfaB homologs (Figure 4, lanes 4 and 5). Although the corresponding Y456 mutation was not tested in PfaBs, its strict conservation suggests an analogous role. Structural analysis of PfaBs revealed a Y456–Q539 hydrogen bond (Figure 5b), reminiscent of the H650–N715 interaction in DynE8‐MAT. This interaction likely contributes to stabilization of the acyl‐enzyme intermediate and protection from water‐mediated nucleophilic attack.

Taken together, these observations indicate that PfaB is not a conventional hydrolase. Hydrolytic activity reported for certain PfaB homologs may result from disruption of the conserved Tyr/Leu‐Gln/Asn hydrogen bond or from conditions that destabilize the acyl‐enzyme intermediate. In this context, hydrolysis may occur only under specific conditions, such as the absence of ACP, rather than representing its primary function.

### 3.4 Evolutionary specialization and substrate accommodation

We propose that PfaB likely arose through gene duplication and subfunctionalization of the PfaA‐KS‐MAT didomain (Figure 1), specializing in the transfer of mid‐ and long‐chain intermediates. In contrast, PfaA‐MAT selects malonyl‐CoA units to perform the initial acyl‐chain elongation (Santín & Moncalián, 2018). Unlike PfaA‐MAT, which retains conserved glutamine and arginine residues required for malonyl‐CoA specificity (Liew et al., 2012; Yadav et al., 2003), bacterial PfaB proteins lack this Gln‐Arg pair (Q678 and R731 in PfaAs‐MAT). Docking predictions suggest that these residues in PfaAs‐MAT sterically clash with long‐chain substrates such as palmitoyl‐CoA (Figure 8a). Substitution of these residues with smaller or acidic amino acids (e.g., I429 and N482 in PfaBs) in PfaB proteins allows accommodation for these substrates (Figure 8b). In addition, structural modeling and docking predictions suggest that PfaB proteins likely accommodate long‐chain fatty acids via a lipophilic tunnel delimited by helices α14‐α17 in PfaBs, a structural feature absent from PfaA‐MAT models.

### 3.5 Product specificity: DHA versus EPA

Sequence analysis reveals conserved motifs differing between DHA‐type and EPA‐type PfaB proteins, notably within helix α16 of PfaBs. Docking models suggest that bulkier residues in EPA‐type PfaBs may sterically limit accommodation of DHA (Figure 8c), whereas DHA‐type PfaBm contains smaller residues compatible with longer chains (Figure 8d). Moreover, AlphaFold3 predicts that L538 in PfaBm acts as a lid interacting with DHA (Figure 8d), this residue is conserved in other DHA‐type PfaB. In contrast, the corresponding residue in PfaBs (E399), also found in other EPA‐type PfaB (Figure S8), is at a distant position to interact. These structural differences provide a plausible structural rationale for product specificity. However, they remain hypothetical and require experimental validation.

### 3.6 In vivo evidence supports a key late‐stage role for PfaB

GC analyses revealed a C18 basal species across all analyzed *E. coli* strains including pDHA4Δ*pfaBm* (Figure S1d). Thus, in the absence of PfaB, no peaks corresponding to longer‐chain PUFA products were detected. Basal expression of *pfaBm* enabled DHA synthesis and complementation of pDHA4Δ*pfaBm* with pET29c::*pfaBm* rescued production. Overexpression of *pfaBm* inhibits DHA synthesis, likely reflecting stoichiometric imbalance within the PUFA synthase complex (Table 1). Consistent with the previous role proposed for PfaB homologs (Almendáriz‐Palacios et al., 2021; Hayashi et al., 2020), PfaBm could hydrolyze intermediates under low abundance of ACP.

Expression of *pfaBs* under IPTG induction shifts the pathway toward EPA production (Table 1). Increased PfaBs abundance compensates for its lower intrinsic efficiency in the *M. marina* system. At basal expression levels, PfaBs disrupt late‐stage PUFA production, leading to accumulation of intermediate species (C18–C20; Figure S1g,h).

Overall, these observations align with earlier reports indicating that PfaB identity determines the final PUFA product (Orikasa et al., 2009).

### 3.7 Functional implications and proposed role

Collectively, these data establish PfaB as a late‐stage acyltransferase essential for PUFA biosynthesis. While PfaC‐KS‐CLF performs the final condensation reactions (Santín et al., 2020), PfaB appears to be required for efficient processing and flux of intermediates beyond C18. A potential, non‐exclusive role for PfaB could be the off‐loading of DHA or EPA to soluble FAS‐ACP for membrane lipid incorporation. Although our in vitro experiments demonstrate only transfer from acyl‐CoA, in vivo the acyl intermediate is covalently attached to the ACP PPT prosthetic group. Because PPT is derived from CoA, the chemistry of inter‐ACP transfer is analogous to acyl‐CoA to ACP transfer, making this mechanism plausible. However, whether this occurs in vivo remains to be confirmed. Alternatively, bound ligands may undergo hydrolysis under certain conditions, such as stress or absence of ACP, consistent with previous reports (Almendáriz‐Palacios et al., 2021; Hayashi et al., 2020).

In conclusion, our structural, biochemical, and in vivo data identify PfaB as a key determinant of PUFA chain length and product identity. The crystal structure of PfaBs and docking analyses provide a framework for understanding substrate specificity. These findings provide a foundation for future mechanistic studies, including quantitative kinetic characterization and for engineering of PUFA synthases.

## 4 METHODS

### 4.1 Strains and culture conditions

*Escherichia coli* strain DH5α was used for molecular cloning and BL21 (DE3) for recombinant protein expression. Cultures were grown in LB medium with the required antibiotics: chloramphenicol (25 μg/mL), kanamycin (50 μg/mL), and/or ampicillin (100 μg/mL) (Sigma‐Aldrich). Solid media contained 1.5% agar.

### 4.2 DNA manipulation and plasmid construction

pDHA4 (Orikasa et al., 2009) was used to amplify *pfaBm* and *ACPm*, which were cloned into pET29c. *pfaE* was instead cloned into pET3a for co‐expression with *ACPm* to obtain holo‐ACP (Santín & Moncalián, 2018). *pfaBs* (NCBI: OZ287685) was amplified from synthetic gene fragments (IDT) and cloned into pET29c (Table S1).

PCR amplifications were performed with Phusion polymerase (Thermo‐Fisher) and custom oligos (Sigma‐Aldrich). PCR products were purified from agarose gels (GenElute, Sigma‐Aldrich) and assembled by Gibson cloning (Gibson et al., 2009). DNA concentrations were measured with a NanoDrop ND‐1000 spectrophotometer (Thermo‐Scientific).

Recombinant constructs were transformed into *E. coli* DH5α via electroporation using 0.2 cm Gene Pulser cuvettes (Bio‐Rad) in a MicroPulser™ electroporator (Bio‐Rad). Colony PCR screening was performed using Biotaq polymerase (Bioline), and Sanger sequencing (Eurofins) was used to verify the construct sequences.

Site‐directed mutagenesis was performed using the QuikChange protocol (Agilent), and PCR products were digested with DpnI restriction enzyme (Thermo‐Fisher).

### 4.3 Fatty acid profile analysis

*Escherichia coli* BL21(DE3) transformed with the corresponding plasmids was grown at 37°C in LB medium. At an OD_600_ of 0.5–0.7, cultures were cooled to 15°C (Amiri‐Jami et al., 2015; Amiri‐Jami & Griffiths, 2010) and protein expression was induced with 0.1 mM IPTG. Cells were harvested at an OD_600_ of 2–4 and pelleted by centrifugation. Cell pellets were frozen at −20°C and sent to Biomar Microbial Technologies (León, Spain) for fatty acid methyl ester (FAME) analysis by gas chromatography with flame ionization detection (FID). Fatty acids were identified by retention time using known standards.

### 4.4 Protein expression and purification

Protein expression was induced at an OD_600_ of 0.6–0.8 with 0.1 mM IPTG, and cultures were incubated at 15–18°C post‐induction. Cells were harvested, frozen at −80°C, and lysed in buffer A (300 mM NaCl, 50 mM Tris–HCl pH 7.5, 20 mM imidazole, 1 mM PMSF, 1 mM DTT). His‐tagged proteins were purified with HisTrap HP columns (Cytiva) and eluted with a linear imidazole gradient (buffer B containing 500 mM imidazole).

PfaBm and PfaBs were further purified by anion exchange chromatography (HiTrap Q, Cytiva) followed by size‐exclusion chromatography (SEC) on a Superdex 200 10/300 GL column in SEC buffer (0.15 M NaCl, 25 mM Tris–HCl pH 7.5, 1 mM EDTA, 1 mM DTT). Proteins were stored at −80°C with 5% (v/v) glycerol.

### 4.5 Radioactive binding assays

Reactions (20 μL) contained ^14^C‐palmitoyl‐ or ^14^C‐malonyl‐CoA (40 Ci/mol; PerkinElmer), PfaBm or PfaBs (20 μM), and ACPm (20 μM) in SEC buffer. After incubation for 45 min at 25°C, reactions were quenched with protein loading buffer (250 mM Tris–HCl pH 6.8, 0.02% w/v bromophenol blue, 10% v/v SDS, 30% v/v glycerol) and loaded onto 10 or 15% SDS‐PAGE gels. After electrophoresis, the gels were stained with Coomassie Brilliant Blue (Sigma‐Aldrich), dried, and exposed for autoradiography.

### 4.6 Liquid chromatography‐mass spectrometry assays

Purified PfaBm and PfaBs (40 μg) were analyzed at the Stanford University Mass Spectrometry Laboratory (Stanford, CA, USA) using an Acquity UPLC system coupled to an SQD2 mass spectrometer (Waters). Separation was performed on an Agilent ZORBAX StableBond 300 C8 column (3.5 μm, 2.1 mm × 50 mm). Intact protein masses of PfaA‐ACPm and its acyl‐ACPm derivatives were analyzed within the mass range of m/z 11,000–12,500 using MaxEnt1 software (Waters).

### 4.7 Protein crystallization and structure determination

PfaBs (16 mg/mL) was crystallized in SEC buffer by vapor diffusion at 25°C using 100 mM HEPES pH 7.5, 8% v/v ethylene glycol (EG), and 10% w/v PEG 8000. Crystals were cryoprotected with 25% EG. Data were collected at ALBA synchrotron (XALOC beamline) and processed with the CCP4 suite (MOSFLM, SCALA) (Battye et al., 2011). The structure was solved by molecular replacement using an AlphaFold3 model (Abramson et al., 2024) with MolRep (CCP4) and refined in Phenix (Adams et al., 2002; Battye et al., 2011). Manual model building was performed in Coot (Emsley & Cowtan, 2004). The final 2.9 Å model was deposited in PDB (ID 9RY8). Structural figures were generated with PyMOL (Schrödinger, LLC.), multiple‐sequence alignments were produced with Clustal‐Omega (Madeira et al., 2024) and rendered with ENDscript (Robert & Gouet, 2014).

### 4.8 PfaB molecular docking

The PfaBs‐ACPs interaction was modeled using AlphaFold3 in monomer mode. The PfaBs crystal structure replaced the predicted model, while theAlphaFold3 predicted model of ACPs was retained (residues 1269–1353 from *S. baltica* PfaA). Docking with palmitoyl‐CoA and DHA‐CoA was performed with SwissDock (AutoDock Vina) (Bugnon et al., 2024; Eberhardt et al., 2021; Grosdidier et al., 2011; Trott & Olson, 2010), ligands were input as SMILES. The search space was centered on the catalytic S457 of PfaBs for palmitoyl‐CoA and on S608 of the PfaBm AlphaFold3 model for DHA‐CoA. The docked PfaBs‐palmitoyl‐CoA complex was structurally aligned with the AlphaFold3 model of PfaAs‐MAT. Similarly, the predicted PfaBm‐DHA‐CoA complex was aligned with the crystal structure of PfaBs to compare the ligand‐binding sites.

## AUTHOR CONTRIBUTIONS

**Nahuel Lofeudo:** Writing – original draft; validation; investigation; formal analysis; data curation; visualization; methodology; writing – review and editing. **Aurora Martín:** Investigation; methodology; visualization; formal analysis. **Mateo Jácome:** Investigation; methodology. **Xia Wan:** Resources; data curation; validation. **María Lucas:** Supervision; writing – review and editing; resources. **Gabriel Moncalián:** Conceptualization; funding acquisition; supervision; resources; writing – review and editing; project administration.

## Supporting information


**Data S1.** Supporting Information.

## Data Availability

The data that support the findings of this study are openly available in Protein Data Bank at https://www.rcsb.org/, reference number 9RY8.
