# Supplementary material for: Prokaryotic PfaB is a terminal acyltransferase that determines the final PUFA product
Source: Protein Sci. 2026 Feb 12;35(3):e70497. doi: 10.1002/pro.70497 (PMC12895367; doi:10.1002/pro.70497)
Supplement: Supplementary file 1 — Data S1. Supporting Information. [file PRO-35-e70497-s001.docx]

**Prokaryotic PfaB is a terminal acyltransferase that determines the final PUFA product**

Nahuel Lofeudo*, Aurora Martín*, Mateo Jácome, María Lucas, Xia Wan and Gabriel Moncalián^a,1^

**SUPPORTING INFORMATION**

Table of contents:

Figure S1_____________________2

Figure S2_____________________3

Figure S3_____________________4

Figure S4_____________________5

Figure S5_____________________6

Figure S6_____________________6

Figure S7_____________________7

Figure S8_____________________8

Figure S9_____________________9

Table S1______________________10


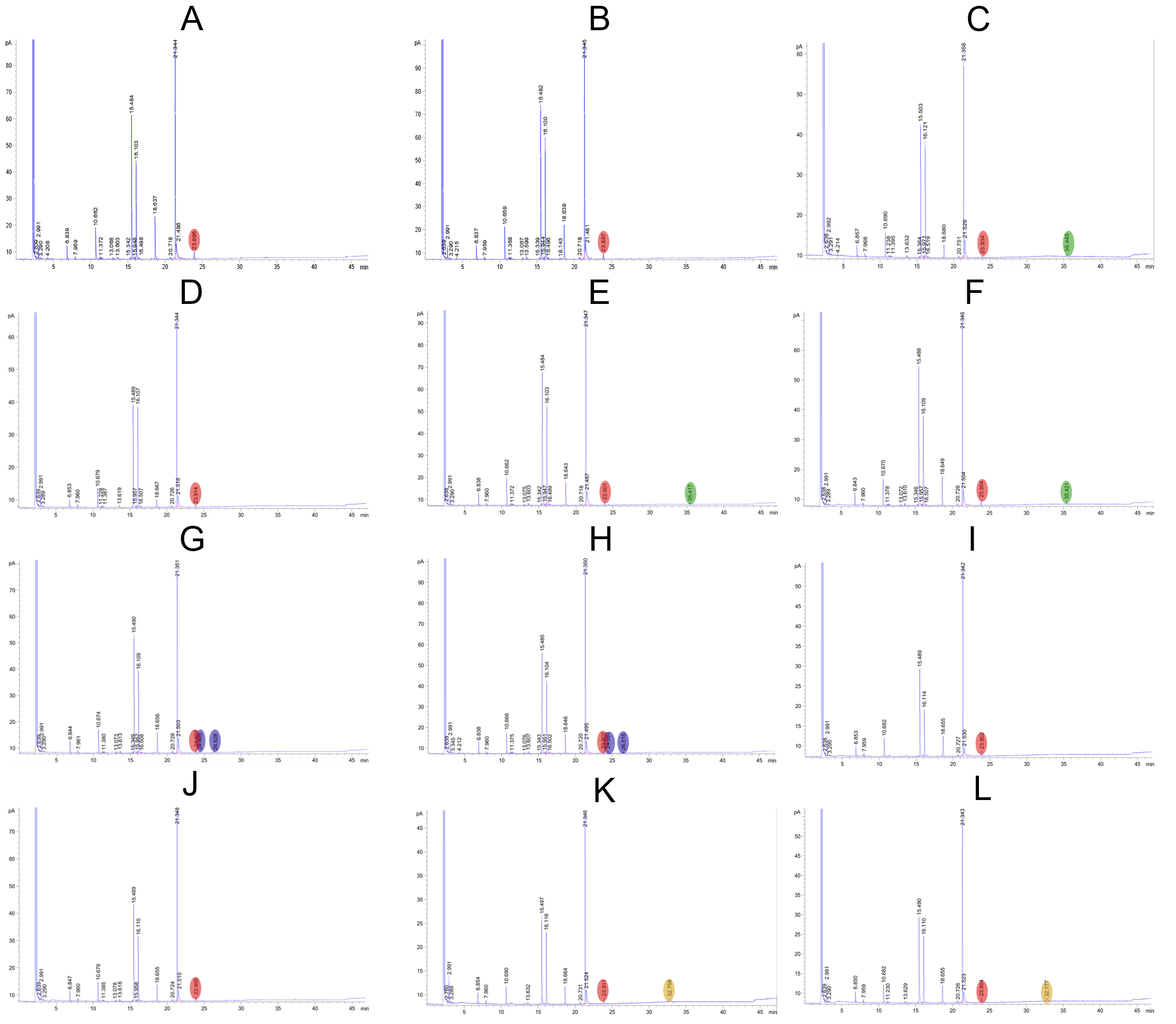


**FIGURE S1. GC-FID analysis of the fatty acid profiles from recombinant *E. coli* BL21(DE3) strains.** The Y-axis represents detector response (pA), and the X-axis shows retention time (min). A peak at 23.9 min (red oval), corresponding to a basal fatty-acid intermediate, is observed in all strains. Additional peaks at longer retention times appear only upon expression of PfaB variants. (A) *E. coli* BL21(DE3) strain, negative control. (B) *E. coli* BL21(DE3) strain incubated with IPTG 0.1 mM. (C) pDHA4, showing a unique DHA peak at 35.4 min (green oval). (D) pDHA4ΔpfaBm, accumulation of the 23.9 min peak and absence of long-chain PUFA products. (E) pDHA4ΔpfaBm +pET29c::*pfaBm*, showing restoration of the DHA peak at 35.4 min (green oval). (F) pDHA4 + pET29c::*pfaBm*, also showing a DHA peak at 35.4 min. (G) pDHA4 + *pfaBs* and (H) pDHA4ΔpfaBm + pET29c::*pfaBs* displaying additional peaks at 24.4 and 26.5 min (blue ovals), corresponding to C18–C20 intermediates. (I) pDHA4 + pET29c::*pfaBm* expressed with IPTG. (J) pDHA4ΔpfaBm + pET29c::*pfaBm* expressed with IPTG. (K) pDHA4 + *pfaBs* and (L) pDHA4ΔpfaBm + pET29c::*pfaBs* expressed with IPTG, showing an EPA peak at 32.7 min (orange ovals).


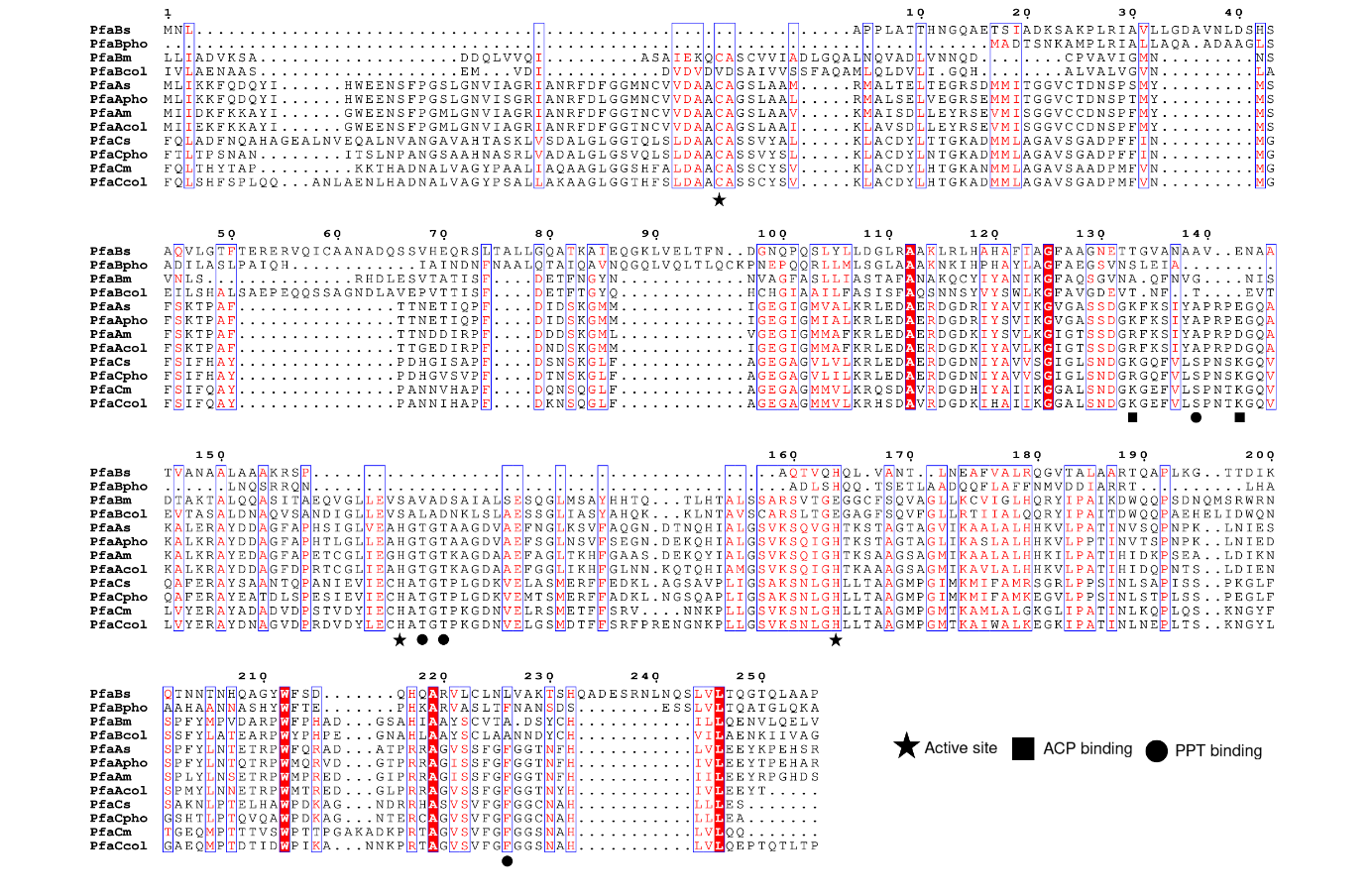


**FIGURE S2. PfaB-KS’ multiple sequence alignment.** Numbering corresponds to PfaBs-KS’. Sequence homology is marked in red; sequence identity is presented with white letters on a red background. The UniProt accession numbers of the proteins used in the alignment are as follows (NCBI entry given when indicated): PfaBs, *S. baltica* PfaB (NCBI: OZ287685); PfaBm, *M. marina* (A0A5J6WK78); PfaBpho, *Photobacterium profundum* (Q93CG7); PfaBcol, *Colwellia* sp. MT41 (A0A0S2JES7); PfaAs, *S. baltica* (NCBI: OZ346655); PfaAm, *M. marina* (A0A5J6WHR2); PfaApho, *P. profundum* (Q93CG8); PfaAcol, *C.* sp. MT41 (A0A0S2JKT5); PfaCs, *S. baltica* (NCBI:  OZ346654); PfaCm, *M. marina* (A0A5J6WI62); PfaCpho, *P. profundum* (Q93CG6); PfaCcol, *Colwellia* sp. MT41 (A0A0S2JER0). The alignment was plotted with ENDscript.

**
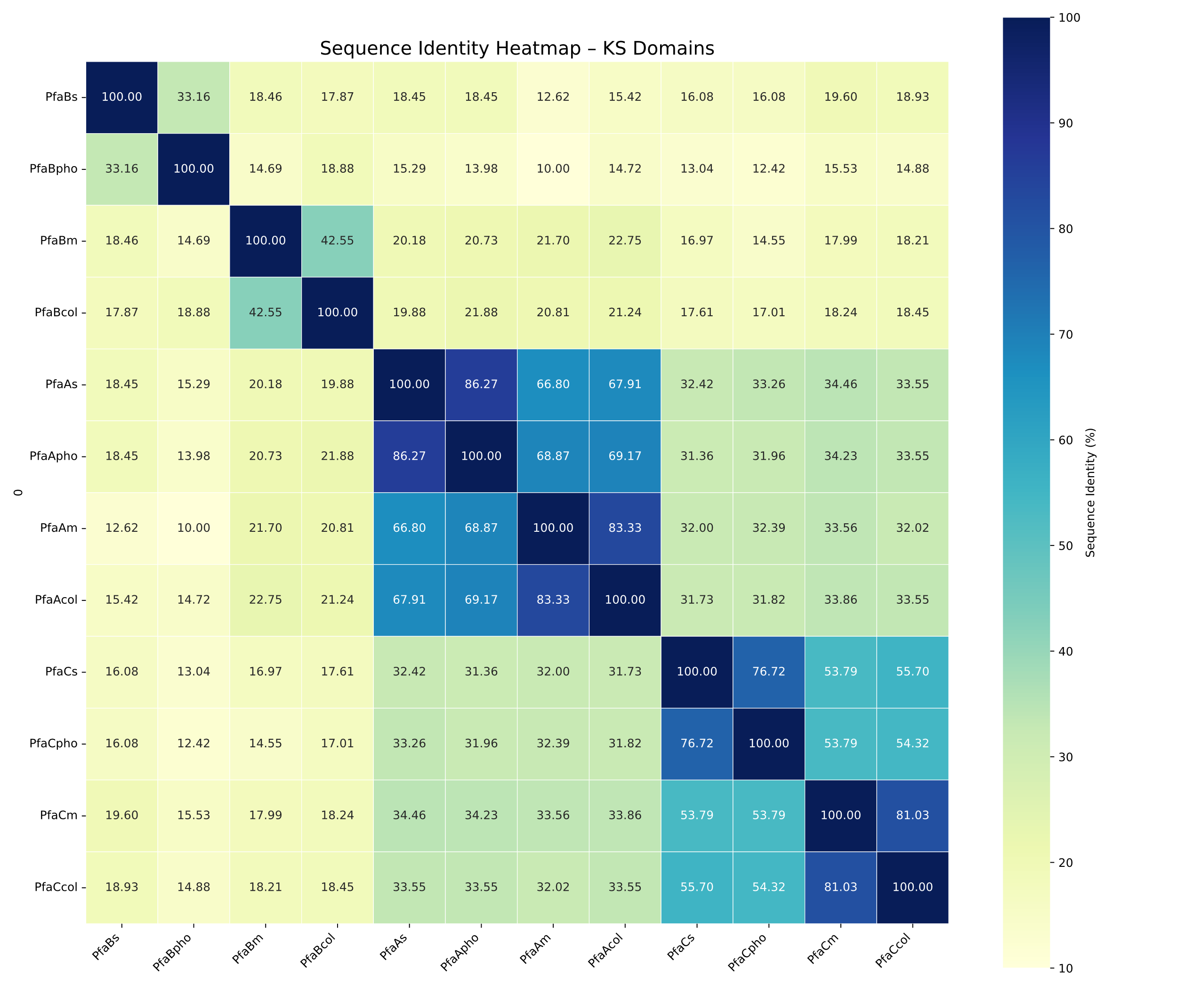
**

**FIGURE S3.** **Heatmap showing pairwise sequence identity (%)** **among KS domains.** The sequences used correspond to those from the MSA shown in Figure S2. Values are derived from Clustal Omega multiple sequence alignments.


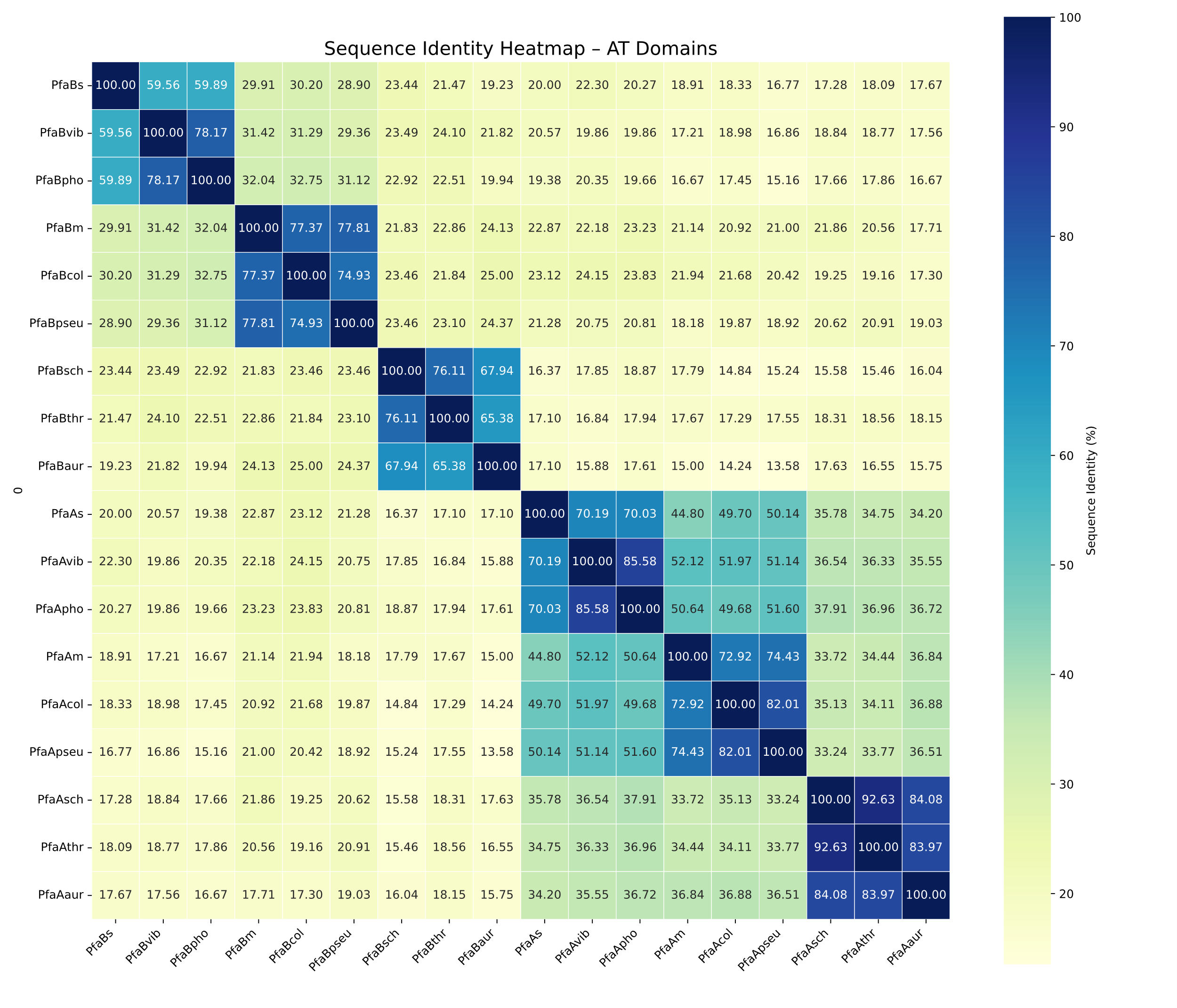


**FIGURE S4.** **Heatmap showing pairwise sequence identity (%)** **among AT domains.** The sequences used correspond to those from the MSA shown in Figure 3. Values are derived from Clustal Omega multiple sequence alignments.


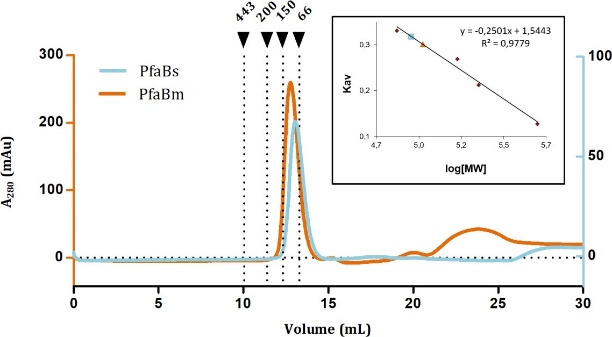


**FIGURE S5. SEC analysis of PfaBs (cyan) and PfaBm (orange).** Left panel: Chromatogram showing UV absorbance as a function of elution volume. SEC was performed using a Superdex 200 10/300 GL column. Molecular weight (MW) standards are indicated: Apoferritin (443 kDa), amylase (200 kDa), alcohol dehydrogenase (150 kDa) and bovine serum albumin (66 kDa). Right panel: Calibration curve plotting the average distribution constant (Kav) versus the logarithm of molecular weight. MW standards are represented as diamonds, PfaBs as a cyan square and PfaBm as an orange triangle. Theoretical MW: 79.736 KDa (PfaBs) and 96.940 kDa (PfaBm). MW obtained from SEC: 79.935 kDa (PfaBs) and 94.452 kDa (PfaBm).


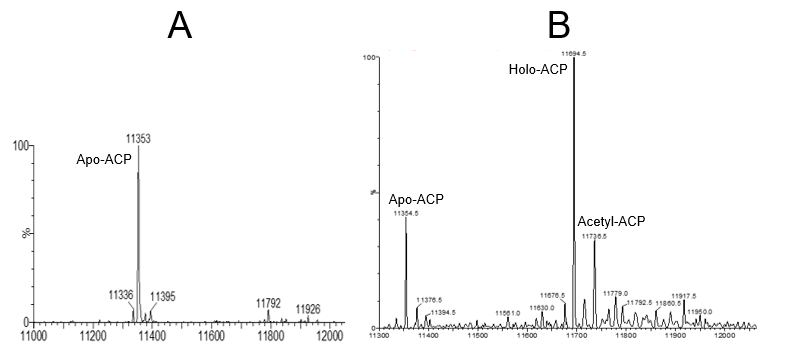


**FIGURE S6. Mass spectrometric analysis of ACP activation.** (A) Mass spectra of the PfaA ACPm domain (PfaA residues 1255–1348) in the apo form following purification by size-exclusion chromatography (SEC). (B) Mass spectra of PfaA-ACPm after co-expression with PfaE and purification by SEC, showing conversion to the holo form. For both samples, 40 µg of protein were analyzed. Spectra were manually acquired over an m/z range of 11000–12500, and deconvolution was performed using default parameters.


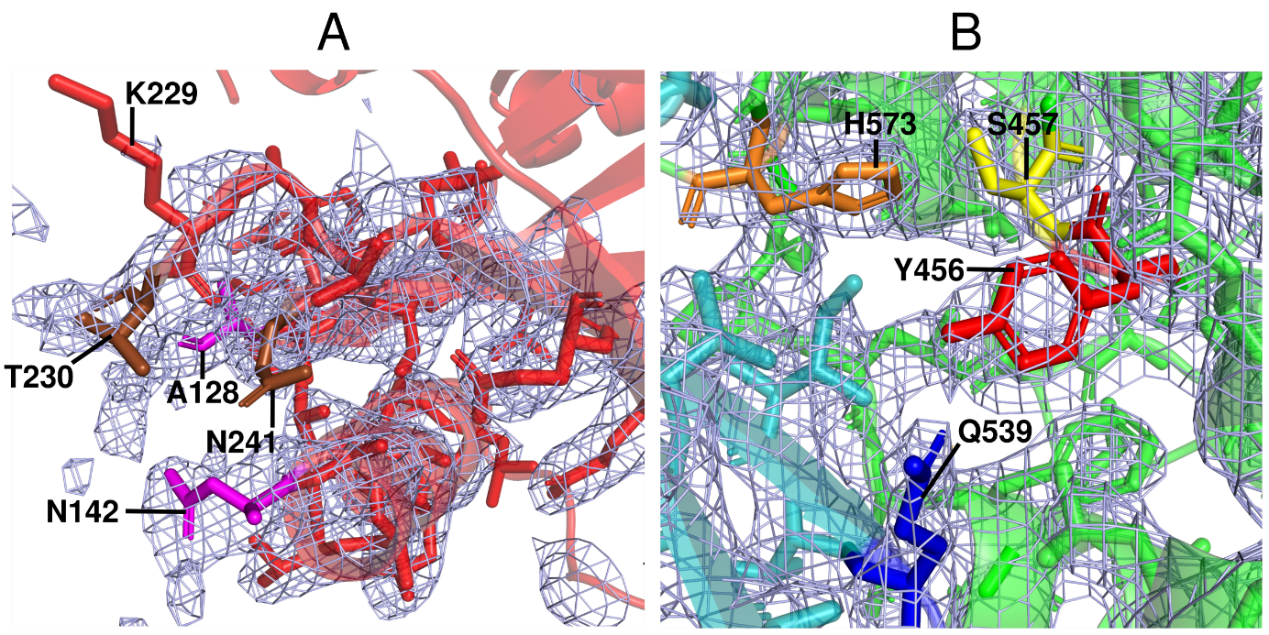


**FIGURE S7.** Representative 2Fo–Fc electron density maps contoured at 1.0 σ. (A) KS′ pseudo-domain of PfaBs shown in cartoon representation (red). Electron density is weak or absent for residues A128–N142 (magenta sticks) and T230–N241 (brown sticks) and K229; indicating local disorder consistent with the higher B-factors observed. (B) Active site of PfaBs. Electron density is well defined around the catalytic residues S457 (yellow sticks) and H573 (orange sticks), as well as for the hydrogen bond formed between Y456 (red sticks) and Q539 (blue sticks).


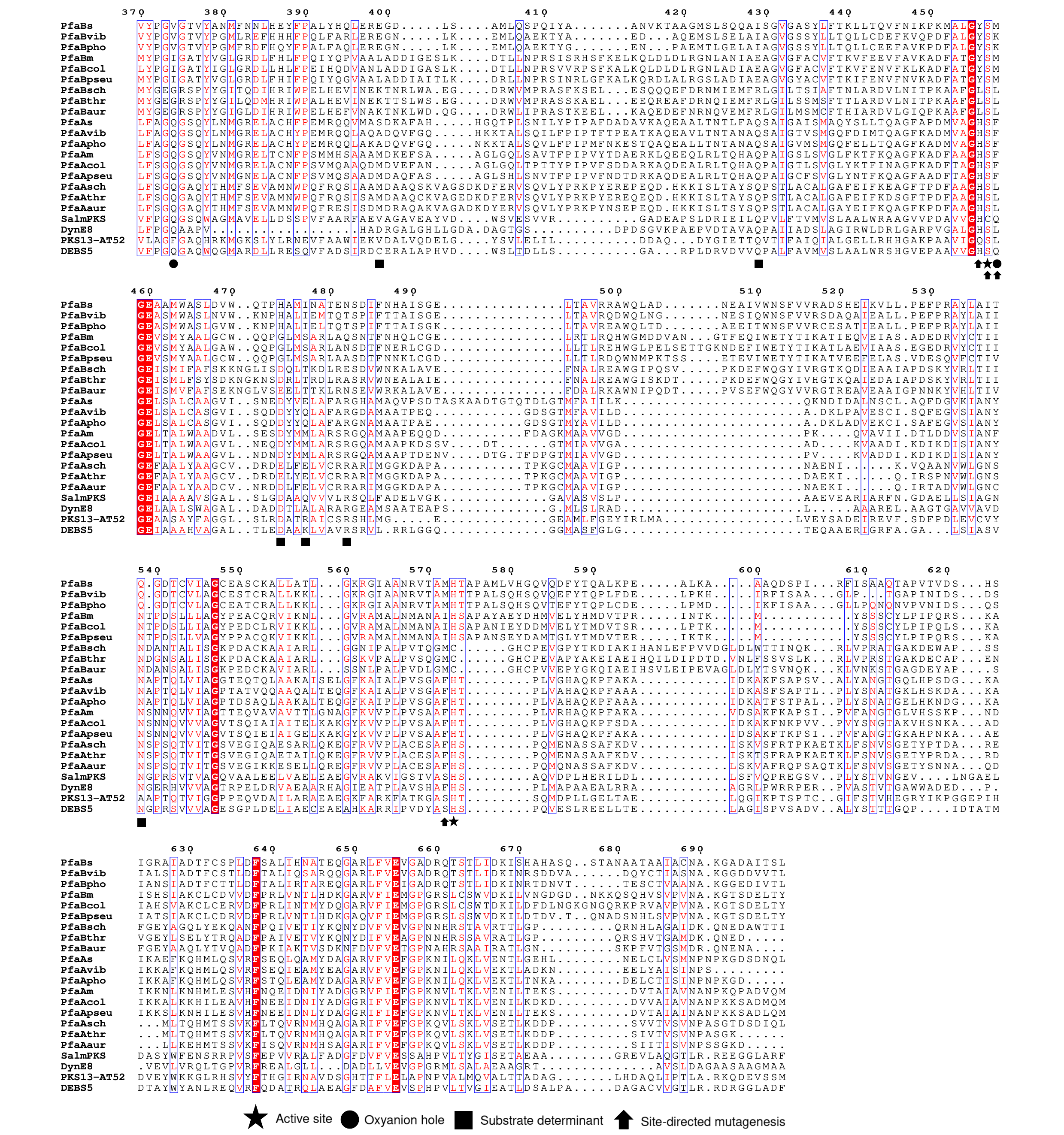


**FIGURE S8. PfaB-AT multiple sequence alignment.** Residue numbering corresponds to PfaBs-AT. Identical residues present in all sequences are shown as white letters on a red background, whereas conserved residues are indicated in red. UniProt accession numbers of the proteins used in the alignment are as follows (NCBI entries indicated where applicable): PfaBs, *S. baltica* PfaB (NCBI: OZ287685); PfaBvib, *Vibrio splendidus* (A0AB35N5F5); PfaBpho, *Photobacterium profundum* (Q93CG7); PfaBm, *M. marina* (A0A5J6WK78); PfaBcol, *Colwellia* sp. MT41 (A0A0S2JES7); PfaBpseu, *Pseudoalteromonas denitrificans* (A0A1I1RM50); PfaBsch, *Schizochytrium* sp. (Q94FB7); PfaBthr, *Thraustochytrium* sp. (A0A1B3PEI8); PfaBaur, *Auriantiochytrium* sp. (A0A7H0U711); PfaAs, *S. baltica* (NCBI: OZ346655); PfaAvib, *V. toranzoniae* (A0A125P5E0); PfaApho, *P. profundum* (Q93CG8); PfaAm, *M. marina* (A0A5J6WHR2); PfaAcol, *C.* sp. MT41 (A0A0S2JKT5); PfaApseu, *P. denitrificans* (A0A1I1REG2); PfaAsch, *Schizochytrium* sp. (Q94FB8); PfaAthr, *Thraustochytrium* sp. (A0A1B3PEI6); PfaAaur, *Auriantiochytrium* sp. (A0A7H0ZVK4); SalmPKS, *Streptomyces albus* (H6D573); DynE8, *Micromonospora chersina* (Q84HI8); PKS13-AT52, *Mycobacterium tuberculosis* (O53579); DEBS5, *Saccharopolyspora erythraea* (Q03133). The alignment was plotted with ENDscript.


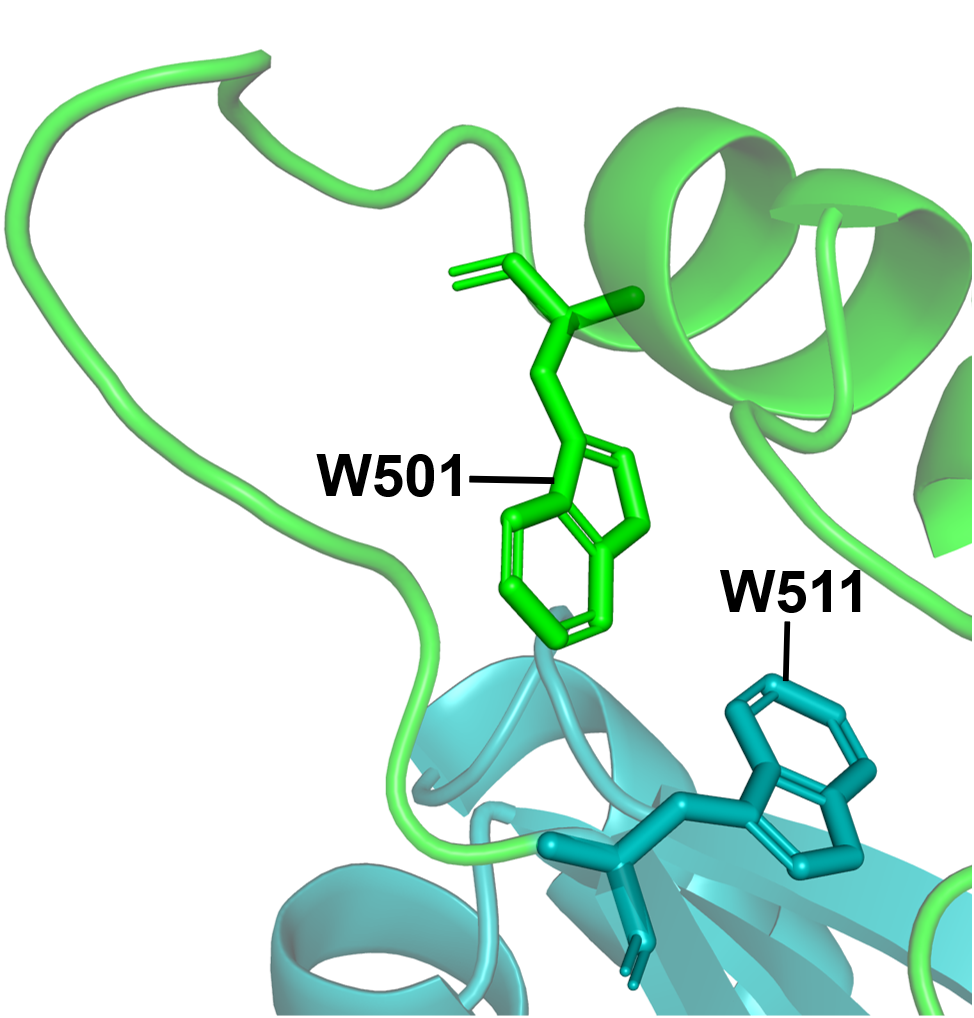


**FIGURE S9.** Stacking interaction between W501 (α/β hydrolase subdomain, green sticks) and W511 (ferredoxin-like subdomain, cyan sticks).

**TABLE S1.** Plasmids used in this work.

| **Plasmid** | **Insert** | **Promoter** | **Size (bp)** | **Resistance** | **Reference** |
| --- | --- | --- | --- | --- | --- |
| **pDHA4** | *pfaA-E* cluster | *M. marina* native | 27,336 | CmR | Orikasa et al. (2009) |
| **pDHA4Δ*pfaB*** | *pfaA-E* with *pfaB* deletion | *M. marina* native | 24,684 | CmR | Derivative from Orikasa et al. (2009) |
| **pET29c::*pfaBm*** | *pfaBm* | T7 | 7,887 | KmR | Santín & Moncalián, (2018) |
| **pET29c::*pfaBm**** | *pfaBm* S608A | T7 | 7,887 | KmR | This work |
| **pET29c::*pfaBm* M1** | *pfaBm* Y607H | T7 | 7,887 | KmR | This work |
| **pET29c::*pfaBm* M2** | *pfaBm* Y607L | T7 | 7,887 | KmR | This work |
| **pET29c::*pfaBm* M3** | *pfaBm* S608C | T7 | 7,887 | KmR | This work |
| **pET29c::*pfaBm* M4** | *pfaBm* M609F | T7 | 7,887 | KmR | This work |
| **pET29c::*pfaBm* M5** | *pfaBm* I728F | T7 | 7,887 | KmR | This work |
| **pET29c::*pfaBs*** | *pfaBs* | T7 | 7,458 | KmR | This work |
| **pET29c::*pfaBs**** | *pfaBs* S457A | T7 | 7,458 | KmR | This work |
| **pET29c::*5ACPm*** | *5ACPm* | T7 | 6,723 | KmR | Santín & Moncalián, (2018) |
| **pET3a::*pfaE*** | *pfaE* | T7 | 5,467 | AmpR | Santín & Moncalián, (2018) |

*PfaBm* refers to *pfaB* from DHA producing *M. marina. PfaBs* refers to *pfaB* from EPA producing *S. baltica.* Vectors used in this work are resistant to chloramphenicol (Cm), kanamycin (Km), or ampicillin (Amp).
